# Functional screening identifies kinesin spindle protein inhibitor filanesib as a potential treatment option for hepatoblastoma

**DOI:** 10.1038/s41698-025-00915-8

**Published:** 2025-04-25

**Authors:** Ruth Nousiainen, Katja Eloranta, Jani Saarela, Antti Hassinen, Tamara J. Luck, Stefano Cairo, Emilie Indersie, Swapnil Potdar, Michaela J. Feodoroff, Jouko Lohi, Lassi Paavolainen, David B. Wilson, Vilja Pietiäinen, Markku Heikinheimo, Marjut Pihlajoki

**Affiliations:** 1 Pediatric Research Center, Children’s Hospital, University of Helsinki and Helsinki University Hospital, Helsinki, Finland; 2 Institute for Molecular Medicine Finland (FIMM), Helsinki Institute of Life Science (HiLIFE), University of Helsinki, Helsinki, Finland; 3 iCAN Digital Precision Cancer Medicine Flagship, University of Helsinki, Helsinki, Finland; 4 XenTech, Evry, France; 5 Champions Oncology, Hackensack, NJ USA; 6 Istituto di Ricerca Pediatrica, Padova, Italy; 7 Department of Pathology, University of Helsinki and Helsinki University Hospital, Helsinki, Finland; 8 Department of Developmental Biology, Washington University School of Medicine, St. Louis, MO USA; 9 Department of Pediatrics, Washington University School of Medicine, St. Louis, MO USA; 10 Faculty of Medicine and Health Technology, Center for Child, Adolescent, and Maternal Health Research, Tampere University, Tampere, Finland

**Keywords:** Paediatric cancer, High-throughput screening, Paediatric cancer, Targeted therapies

## Abstract

Hepatoblastoma is a rare pediatric liver malignancy usually treated with surgery and chemotherapy. To explore new treatment options for hepatoblastoma, drug screening was performed using six cell models established from aggressive hepatoblastoma tumors and healthy pediatric primary hepatocytes. Of the 527 screened compounds, 98 demonstrated cancer-selective activity in at least one hepatoblastoma model. The kinesin spindle protein (KSP) inhibitor filanesib was effective in all models and was further evaluated. Filanesib induced G2/M arrest and apoptosis in hepatoblastoma cells at concentrations tolerable to primary hepatocytes. Prominent nuclear fragmentation was observed in filanesib-treated hepatoblastoma cells. Genes participating in cell cycle regulation were noted to be differentially expressed after filanesib treatment. Filanesib reduced the rate of tumor growth in 4/5 hepatoblastoma mice models. One of these models showed complete growth arrest. Our results suggest that filanesib is a potential candidate for hepatoblastoma treatment and should be investigated in future clinical trials.

## Introduction

Hepatoblastoma (HB) is the most common type of pediatric liver malignancy. The annual incidence is 1.5–2.2 cases per million and is slowly rising^1,2^. Low birth weight and preterm birth are known risk factors of HB^3^. Genetic risk factors include familial adenomatous polyposis (*APC*), Beckwith–Wiedemann syndrome (hemihypertrophy), and Li–Fraumeni syndrome (*TP53*)^4–7^. HB is usually diagnosed in children under 5 years^8^. Current European standard-of-care treatment protocols are based on the International Society of Paediatric Oncology Epithelial Liver Tumor Group (SIOPEL) trials and depend on the risk stratification of disease^9^. Treatment usually entails a combination of surgery and chemotherapeutics, and cure can only be achieved with surgical intervention. First-line chemotherapeutic treatment consists of preoperative and postoperative administration of cisplatin, carboplatin, and doxorubicin. Liver transplantation is an option for unresectable HB. Currently, there is no standard-of-care treatment for relapsed or refractory HB, and 5-year overall survival is less than 50% in this patient group^10^.

Conventional chemotherapeutics pose risks to young patients. Cisplatin treatment is associated with a significant risk of hearing impairment^11^. Anthracyclines, such as doxorubicin, are known to be cardiotoxic and can cause heart failure years after the initial exposure^12^. In principle, targeted drugs and personalized therapies could result in fewer adverse effects; however, such treatments have not been widely used in HB. Exploitation of mutation-targeting therapies has proven difficult because the mutation frequency in HB is low^13^. An exception is *CTNNB1*, a gene encoding β-catenin, which is known to have a high mutation frequency in HB^14^. However, drugs targeting Wnt/β-catenin signaling have shown little potential, and no HB trials have been conducted using these agents^15^. Drug screening is a useful tool to discover new potential treatment modalities, but only one extensive drug screening with three-dimensional (3D) HB models has been published. Kluiver et al. screened HB organoids with over 200 compounds and identified sensitivity to histone deacetylase (HDAC) inhibitors across HB subtypes^16^.

This study aimed to identify new potential compounds with an acceptable toxicity profile for the management of HB and especially for high-risk HB tumors. We used six previously established HB models derived from aggressive tumors and healthy pediatric primary hepatocytes, all cultured as spheroids, to perform an extensive drug screen with over 500 compounds. To validate and further assess the effects of the potential drugs, both in vitro and in vivo experiments were conducted.

## Results

### Characteristics of HB PDX models

Cancer tissue samples for the PDX models were obtained from high-risk HB patients. The cell models used in this study (*n* = 6) were established from the PDX by XenTech (Evry, France)^17^. Clinical information and basic demographics of the models are shown in Supplementary Table 1. Two models were derived from the same patient (HB-279 from primary tumor resection, HB-284 from intraperitoneal metastasis). Of the six HB models, five carry *CTNNB1* mutations.

### Overview of the drug screen and identification of effective drugs

**Fig. 1:**
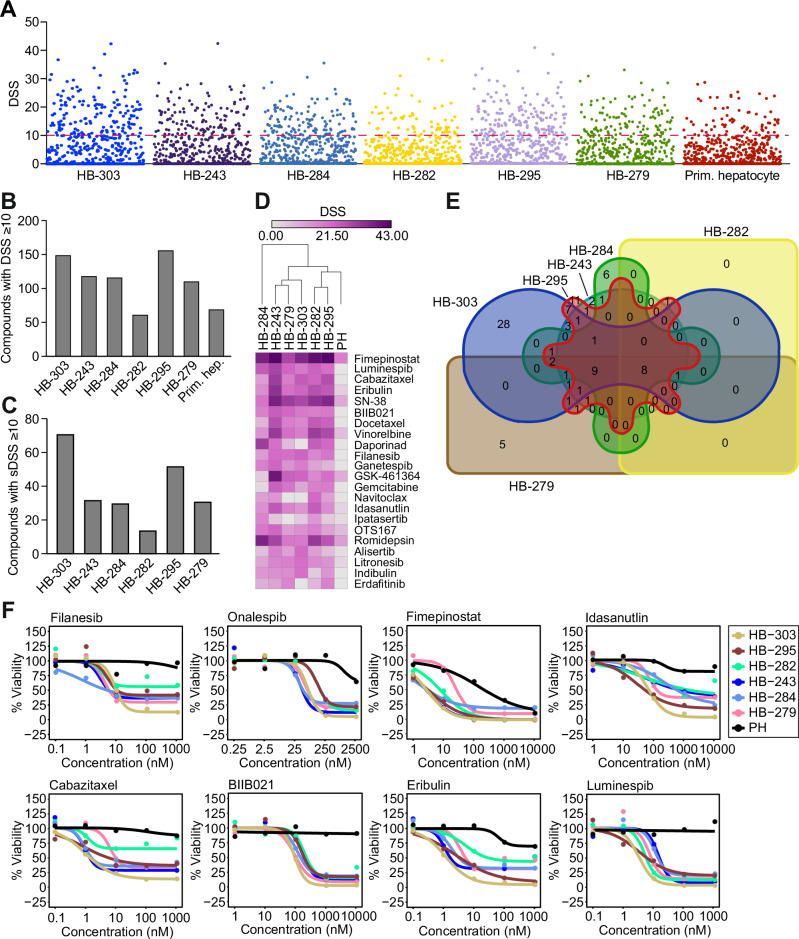
Overview of the results of the high-throughput drug screen. Efficacy of tested drugs in six HB models grown as spheroids was evaluated using DSS. DSS ≥ 10 was considered effective (dotted red line) (**A**). The number of compounds with DSS ≥ 10 (**B**). Taking into account the effect of the drug on healthy primary hepatocytes, sDSS was calculated. Drugs with sDSS ≥ 10 were considered effective and non-toxic (**C**). The top 10 compounds with the highest sDSS of each cell line are shown (**D**) intensity of color depicts DSS. Venn diagram shows overlapping compounds with sDSS ≥ 10 (**E**). The dose–response curves of these eight overlapping compounds are shown in panel **F**. DSS drug sensitivity score, sDSS selective drug sensitivity score, PH primary hepatocytes.

The drug screen consisted of 528 approved or emerging oncological compounds and combinations (527 individual drugs, one combination) (Supplementary Table 2). The screened compounds have varying mechanisms of action, including both agents with a specific target as well as conventional chemotherapeutics (Supplementary Fig 1A). Z-factors were calculated to assess the robustness of the screen^18^. Z-factors of >0.5 were found for all screens, demonstrating sufficient quality (Supplementary Fig 1B). Drug sensitivity score (DSS) was calculated for each tested compound, as previously described^19^. A DSS ≥ 10 was considered effective. General drug sensitivity varied between the HB cell models (Fig. 1A). HB-303 and HB-295 cells, representing fetal HB, had the highest number of hits considered effective, 149 (28%) and 157 (30%), respectively (Fig. 1B). The other four HB cell models, with predominantly embryonal histology, demonstrated responses to 62–119 compounds with DSS ≥ 10 (Fig. 1B). Seventy out of 527 drugs (13%) also reduced the viability of control primary hepatocytes at this cut-off (Fig. 1A, B).

### Ninety-eight compounds reduced HB cell viability while being non-toxic to normal hepatocytes

To evaluate the tumor-selective effect of the drugs, compounds toxic to the age-matched healthy primary hepatocytes were excluded by calculating a selective drug sensitivity score (sDSS). The list of potential candidate compounds was narrowed down using a sDSS ≥ 10 as a cutoff (Fig. 1C). The highest-scoring compounds included epigenetic and metabolic modifiers, mitotic inhibitors, modulators of apoptosis, protein kinases, and heat shock protein (Hsp) inhibitors (Fig. 1D). The conventional compounds in HB treatment (carboplatin, doxorubicin, cisplatin, irinotecan, vincristine, and etoposide) demonstrated only a minor effect (Supplementary Fig 2), though it should be noted that all PDX models were established from pre-treated patient tissues (Supplementary Table 1).

After the above-described exclusion criteria were applied, 98 compounds with sDSS ≥ 10 in at least one of the HB cell models remained (Fig. 1C). Eight compounds were found to be effective in all six HB models (Fig. 1E). These were filanesib (kinesin spindle protein [KSP] inhibitor), onalespib (Hsp inhibitor), fimepinostat (PI3K/HDAC inhibitor), idasanutlin (MDM2-antagonist), cabazitaxel (microtubule inhibitor), BIIB021 (Hsp inhibitor), eribulin (microtubule inhibitor), and luminespib (Hsp inhibitor) (Fig. 1E, F). While all eight compounds met the criteria of sDSS ≥ 10, fimepinostat and eribulin showed some toxicity in healthy control cells even at lower concentrations and were thus excluded. Response to both cabazitaxel and idasanutlin was observed across the models. The remaining four compounds (filanesib, onalespib, BIIB021, and luminespib) consisted of three Hsp inhibitors and one KSP inhibitor. Of these, the KSP inhibitor filanesib and one Hsp inhibitor, luminespib, were chosen for further investigation.

### Filanesib and luminespib reduced cell survival and triggered apoptosis in HB spheroids

**Fig. 2:**
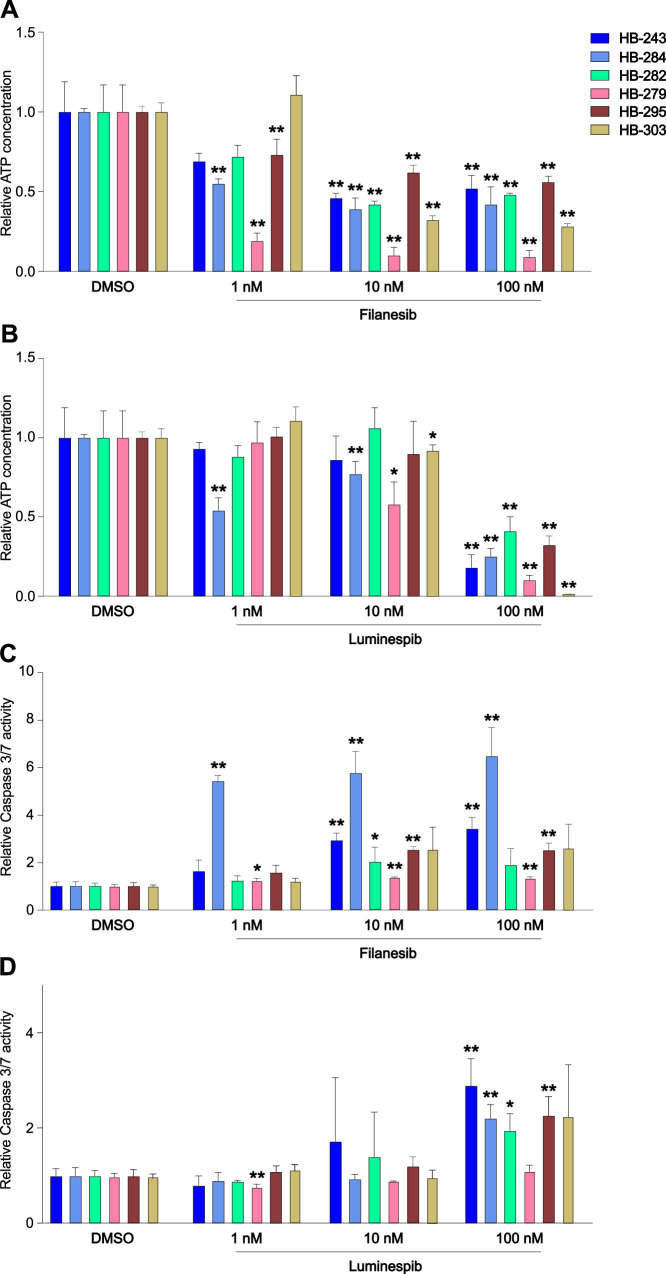
Cell viability and apoptosis in HB spheroids treated with filanesib or luminespib. In contrast to DSRT, spheroids were first established for 72 h and after that chosen compounds were administered. Cell viability was measured by relative ATP concentration and decreased in all cell models after 72 h of filanesib treatment (**A**). Luminespib exposure decreased spheroid survival notably with the highest tested dose (**B**). While filanesib decreased cell viability in all HB models, the mechanism of cell death seemed to be partly caspase 3/7 mediated but varied among the models (**C**). Luminespib increased caspase 3/7 activity in a dose-dependent manner (**D**). * *p* < 0.05, ** *p* < 0.01 (Student’s *t*-test), (*N* = 4).

In the initial screen, cells were seeded on pre-drugged U-bottom plates, where they formed spheroids for 72 h under drug treatment. To simulate the treatment of existing tumors and validate the results, the cells were first allowed to form spheroids and then treated with either filanesib or luminespib. The concentration of ATP was decreased at least 30% in all cell models after 10 or 100 nM filanesib treatment. HB-279 showed an especially strong response to filanesib, with the ATP-concentration decreasing 80% already with 1 nM of filanesib (Fig. 2A). Luminespib-treated HB models showed remarkable, 60–99% decrease in viability with the highest 100 nM concentration (Fig. 2B). Lower luminespib concentrations of 1 nM and 10 nM were less effective.

The magnitude of apoptotic cell death after treatment was assessed using a caspase 3/7 assay. HB-284 showed the highest activation of caspase 3/7 activity after filanesib treatment (Fig. 2C). Interestingly, while filanesib-treated HB-279 cells showed the greatest decrease in cell viability, caspase 3/7 activity did not increase remarkably, suggesting an alternative method of cell death (Fig. 2C). Luminespib-treated models showed increased caspase 3/7 activity in a dose-dependent manner (Fig. 2D).

### Filanesib and luminespib caused alterations in cell morphology

**Fig. 3:**
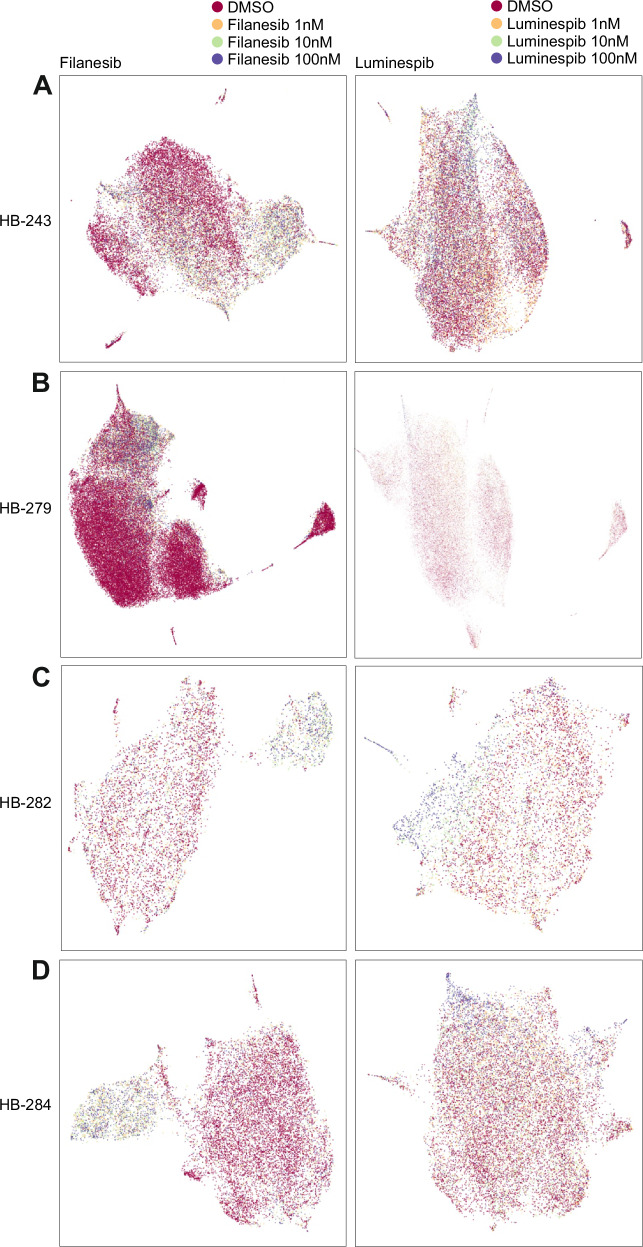
Characterization of morphologic image-based cell features with Cell Painting. UMAPs of all morphological features of four HB models showing nonlinear 2D projection of 300-dimensional single cell data (**A**–**D**). Each dot represents one cell, and cells were grouped based on the similarity of morphological features. Cell numbers vary between models due to cell size.

**Fig. 4:**
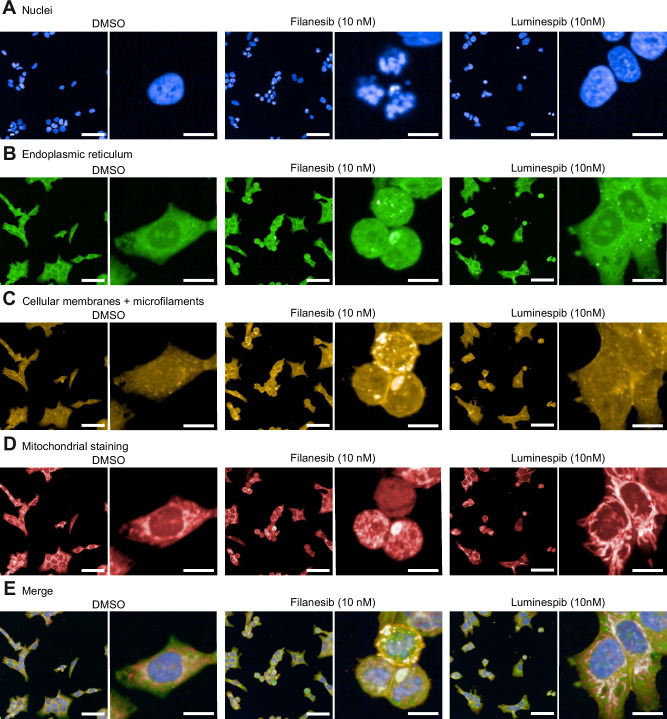
Cell Painting reveals filanesib-treated cells to show signs of mitotic catastrophe. Representative images of HB-279 cells treated with either 10 nM filanesib, 10 nM luminespib, or control (DMSO) (**A**–**E**). Four different wave lengths were used to capture the morphological features. Different stains were used to visualize the nuclei (**A**) endoplasmic reticulum (**B**) cellular membranes and microfilaments (**C**) and mitochondria (**D**). Merged images are presented in (**E**). The nuclei of filanesib-treated cells displayed a fragmented morphology (**A**). Scale bar = 50 µm, scale bar in close-up images = 10 µm.

Cell Painting, a phenotypic, morphological profiling assay for cellular components/organelles was used to visualize the morphological changes caused by filanesib and luminespib treatments. First, UMAPs were used to show a nonlinear 2D projection of 300-dimensional single-cell data with each dot representing one cell, where cells grouped based on the similarity of their morphological features. Filanesib-treated cells clustered by dose and separately from the DMSO controls (Fig. 3A–D). Similarly, luminespib-treated cells clustered together especially at the highest concentration of 100 nM (Fig. 3A–D). In HB-279 cells treated with 10 nM filanesib, the cells became round (Fig. 4A-E), and the nuclei appeared fragmented (Fig. 4A) resembling mitotic catastrophe. This was also observed in HB-284 cells (Supplementary Fig 3C). In HB-243 and HB-282 cells, the fragmentation of nuclei was not as distinct (Supplementary Fig 3A, B). Luminespib induced changes in the mitochondrial morphology (Fig. 4D, Supplementary Fig 3A–C). The lowest (1 nM) concentration of filanesib or luminespib did not cause marked changes in cell morphology (Supplementary Fig 3A–C). The most notable changes caused by filanesib were those in the nuclei shape. Heatmap and clustering of the features (averages, normalized to the controls of each cell line) showed that, in general, samples clustered according to their treatment (Supplementary Fig 4).

### Filanesib caused G2/M cell cycle arrest in HB cells

**Fig. 5:**
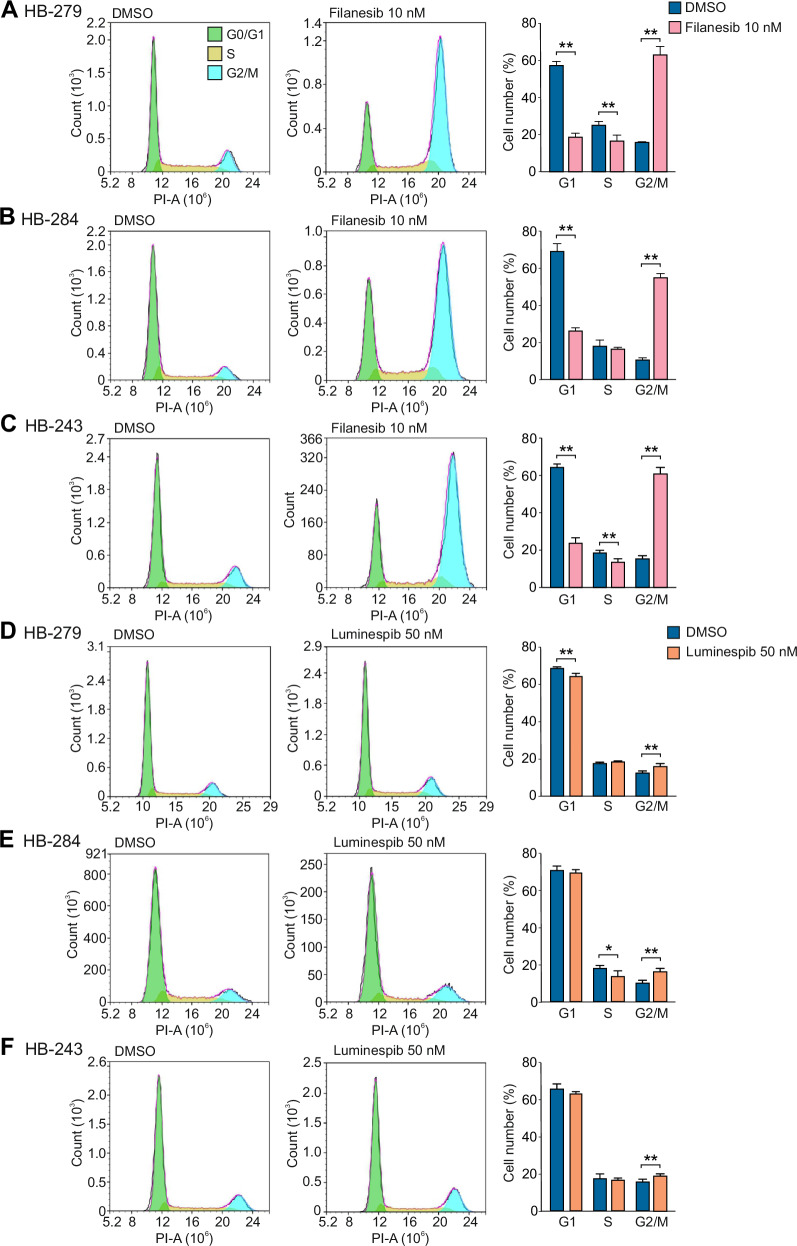
Filanesib and luminespib induce G2/M cell cycle arrest in HB cells. Representative histograms of flow cytometry analysis after 10 nM filanesib treatment of HB-279 cells (**A**) HB-284 cells (**B**) and HB-243 (**C**) for 24 h. Representative histograms of flow cytometry analysis after 50 nM luminespib treatment of HB-279 cells (**D**) HB-284 cells (**E**), and HB-243 cells (**F**) for 48 h. Bar plots show the percentage of cells in each phase of the cell cycle of four independent experiments presented as mean ± SD. ** *p* < 0.01 (Student’s *t*-test).

To investigate the effect of the candidate drugs on cell cycle, HB-279, HB-284, and HB-243 cells were treated with 10 nM filanesib for 24 h or 50 nM luminespib for 48 h or with DMSO. Next, the cell cycle was analyzed using flow cytometry. We found that treatment with filanesib induced a significant increase in the percentage of cells in the G2/M phase in all cell lines (Fig. 5A–C). In DMSO treated HB-279 cells, 16% (SD = 0.2) of the cells were in G2/M phase while in filanesib treated cells, the percentage of G2/M cells was 64% (SD = 4.4) (Fig. 5A). In HB-284 cells, the corresponding percentages were 27% (SD = 0.9) (DMSO) and 56% (SD = 1.9) (filanesib) (Fig. 5B), and in HB-243 cells 16% (SD = 1.1) (DMSO) and 61% (SD = 2.9) (filanesib) (Fig. 5C). Luminespib also caused an increase in the percentage of cells in the G2/M phase, even though the effect was milder than with filanesib (Fig. 5D–F). In DMSO treated HB-279 cells the percentage of cells in the G2/M phase was 13% (SD = 0.6) while 16% (SD = 1.1) of luminespib treated cells were in G2/M phase (Fig. 5D). The corresponding percentages in HB-284 cells were 10% (SD = 1.0) (DMSO) and 17% (SD = 1.2) (luminespib) (Fig. 5E), and in HB-243 cells 16% (SD = 1.0) (DMSO) and 19% (SD = 0.7) (luminespib) (Fig. 5F).

### Silencing *KIF11* caused apoptosis and G2/M cell cycle arrest in HUH6 HB cells

**Fig. 6:**
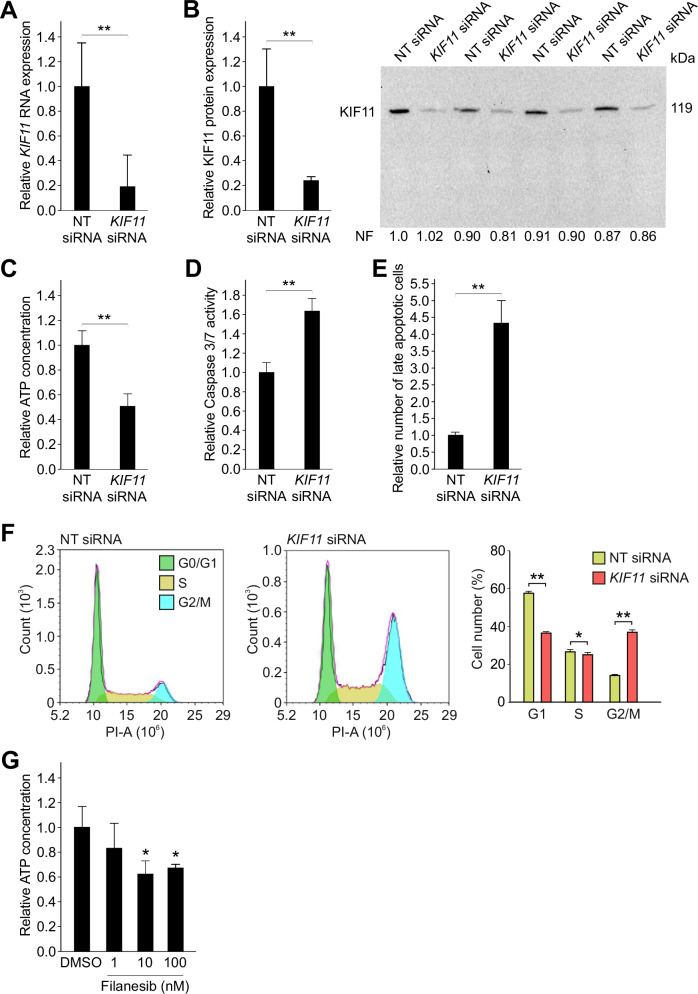
*KIF11* silencing promotes apoptosis and G2/M cell cycle arrest in HUH6 cells. In HUH6 cells, *KIF11* mRNA expression was reduced 80% after 72 h of *KIF11* siRNA transfection compared to NT control (**A**). Protein band intensity of KIF11 was 80% lower in *KIF11* siRNA treated cells in contrast to NT control cells (**B**). ATP concentration decreased 50 % (**C**), caspase 3/7 activity increased 1.6-fold (**D**), and the number of late apoptotic cells increased 4.3-fold (**E**) after KIF11 silencing. *KIF11* silencing induces G2/M cell cycle arrest in HUH6 cells. Representative histograms of flow cytometry analysis after NT siRNA or *KIF11* siRNA transfection (**F**). Bar plots show the percentage of cells in each phase of the cell cycle (**F**). Filanesib reduced HUH6 cell viability measured by relative ATP concentration after 72 h treatment (**G**). Bar plots are presented as relative values of mean ± RSD. Band intensity is normalized to total protein expression of each lane. Normalization factor (NF) describing the amount of total protein in lane in relation to other lanes is given beneath the bands (**B**). **p < 0.01 (relative to DMSO control). NT non-targeting.

To validate the significance of KIF11, the target protein of filanesib, in HB pathobiology the impact of KIF11 on HB cells was assessed by silencing it in HUH6 HB cells. Silencing efficiency was verified at both RNA and protein level. Following transient siRNA transfections, KIF11 expression was reduced 80% at the mRNA and protein level in HUH6 cells (Fig. 6A, B). Next, we assessed the effect of *KIF11* silencing on HB cell viability and apoptosis. *KIF11* silencing caused a 50% reduction in cell viability (Fig. 6C). Apoptosis was assessed with two different methods. *KIF11* silencing resulted in 1.6-fold increase in Caspase 3/7 activity (Fig. 6D) and a 4.3-fold increase in the number of late apoptotic cells (Fig. 6E). Furthermore, the cell cycle analyses revealed that *KIF11* silencing promotes G2/M cell cycle arrest in HUH6 HB cells, mirroring the effect of filanesib treatment (Fig. 6F). Finally, the impact of filanesib on HUH6 cell viability was assessed. Both 10 and 100 nM filanesib caused ∼40% reduction in HUH6 cell viability (Fig. 6G).

### Filanesib decreased HB growth in vivo

**Fig. 7:**
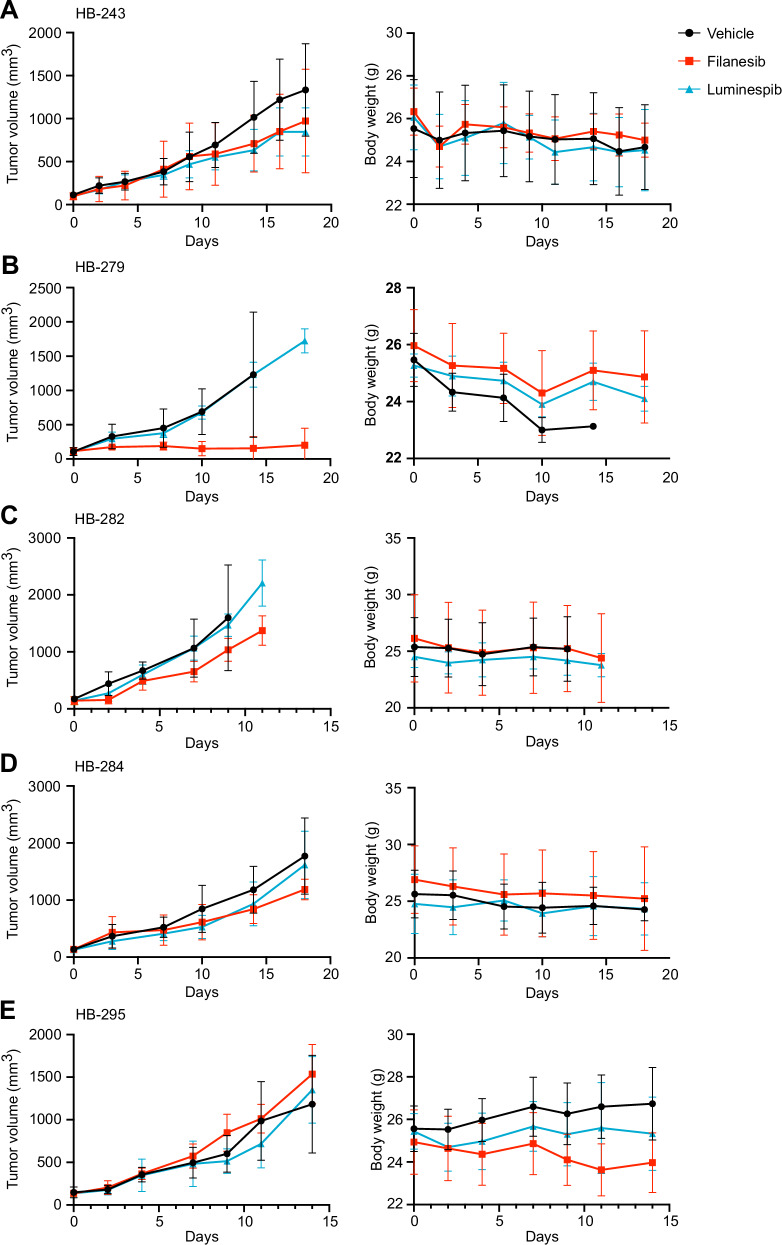
Filanesib reduces HB tumor growth rate in vivo. Changes in tumor volume and bodyweight of mice treated with oral 20 mg/kg filanesib every fourth day (red squares), 30 mg/kg luminespib three times per week (blue triangles) or with vehicle (black circles) are shown (left panel, **A**–**E**). Each point is the mean ± SEM. Each of the five study arms included three mice. Bodyweights are shown in right panel (**A**–**E**).

To assess the efficacy of filanesib and luminespib in vivo, we used five HB PDXs. Mice with HB tumors were randomly assigned to receive either vehicle, filanesib, or luminespib. Tumors treated with vehicle grew rapidly (Fig. 7A–E). Filanesib reduced the growth of tumor volume in 4/5 PDX tumors (Fig. 7A–D). Of these, HB-279 showed a distinctively strong response to filanesib (Fig. 7B). The tumor volume of HB-279 did not increase substantially in any of the filanesib-treated mice during the 18-day treatment period. HB-295 did not show any response to filanesib (Fig. 7E). Only one PDX model, HB-243, showed reduction in tumor growth when treated with luminespib (Fig. 7A). Mice that responded to the treatments did not show a significant change in body weight (Fig. 7A–E, right panel).

**Fig. 8:**
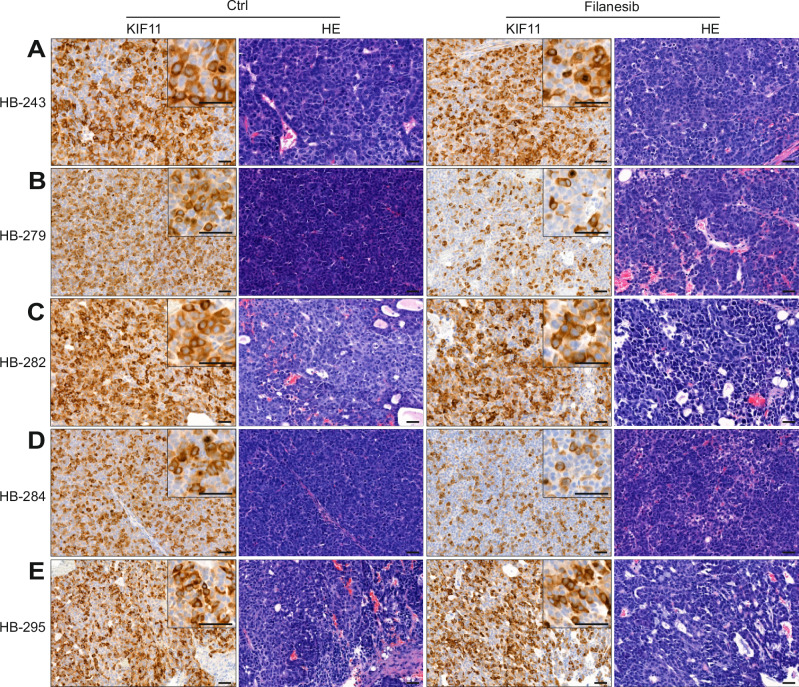
Level of KIF11 protein expression in filanesib or vehicle treated murine tissues. KIF11 expression in HB PDX FFPE tissue samples was evaluated by immunohistochemistry. Baseline KIF11 expression was high in all samples (**A**–**E**). A decrease in KIF11 expression after filanesib treatment was evident in 2/5 HB PDX tumors, HB-279 (**B**) and HB-284 (**D**). H&E-staining shows the histology of the tumors (**A**–**E**). Scale bar = 50 μm. Close-ups in the corners: scale bar = 50 μm.

Tumor tissues resected from filanesib-treated mice were immunostained with KIF11, a target protein of the compound. Hematoxylin and eosin (H&E) staining was performed to show the histology of the tumors (Fig. 8A–E). All untreated tumor tissues showed relatively high expression of KIF11, and the staining was mainly localized in the cytoplasm (Fig. 8A–E). In dividing cells showing mitotic spindle formation, expression was also localized in the nuclei (Supplementary Fig 5A–E). In HB-279 and HB-284 tumors, the number of KIF11-positive cells was lower in filanesib-treated tumor tissues than in controls (Figs. 8B–D).

### Expression of *KIF11* in RNA sequencing data

**Fig. 9:**
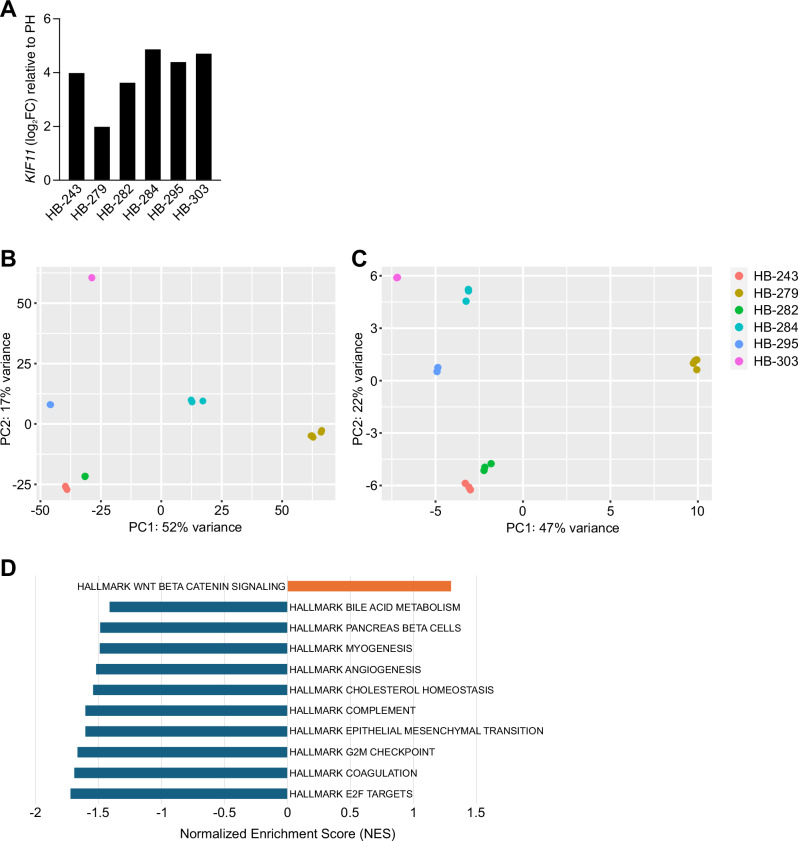
RNA-sequencing profile of HB-279 differs from other HB models. *KIF11* RNA expression in six PDX cell models relative to primary hepatocytes is shown in panel **A** Unsupervised principal component analysis of the expression of the most variably expressed genes (**B**) and of a subset of mitotic spindle-related genes (**C**) shows the relation of untreated HB-279 cells to the other HB models. Each dot represents a single replica sample. Gene set enrichment analysis comparing the RNA-sequencing results of HB-279 to the other models using Hallmark gene sets was done. **D** shows the top 10 highest and lowest scoring Hallmark gene sets with FDR < 0.25. PH primary hepatocytes, PC principal component, NES normalized enrichment score.

To assess the mRNA expression of *KIF11*, RNA sequencing of the six HB models was performed. *KIF11* was overexpressed in all six HB PDX models at mRNA level compared to healthy hepatocytes (Fig. 9A). Five models showed notable *KIF11* overexpression varying between 3.61 and 4.70 log_2_FC, while the overexpression in HB-279 was the lowest at 1.97 log_2_FC (Fig. 9A).

### HB-279 has lower expression of mitotic spindle-related genes compared to the other models

To investigate why HB-279 was more sensitive to filanesib compared to the other models, differentially expressed genes between HB-279 and the other five HB models were analyzed. In total, 729 genes were significantly upregulated and 1819 were downregulated (adjusted p ≤ 0.1). Unsupervised principal component analysis (PCA) showed that HB-279 differed from the other models and that HB-284, the metastasis of HB-279, clustered between HB-279 and the other models (Fig. 9B). When only looking at Hallmark Mitotic Spindle genes, HB-279 clustered even further from the other models (Fig. 9C).

Gene set enrichment analysis (GSEA) was performed to identify enriched gene sets among the differentially expressed genes between HB-279 and other HB models. From the Hallmark gene set collection, 18 gene sets with a false discovery rate (FDR) ≤ 0.25 were found, of which 17/18 had lower expression in HB-279 than in the other models (Fig. 9D). The most negatively enriched gene set was Hallmark E2F Targets, in which the genes had a consistently lower RNA expression in HB-279 than in the other HB models. (Supplementary Fig 6; FDR < 0.005). This gene set includes 200 genes encoding cell cycle -related targets of the E2F transcription factors^20^. It includes four KIF genes (*KIF18B*, *KIF22*, *KIF2C*, and *KIF4A*) but not the filanesib target *KIF11*. The only significantly positively enriched Hallmark gene set in HB-279 vs. other HBs was Wnt/β-catenin signaling (Fig. 9D).

Next, expression of the Hallmark Mitotic Spindle gene set was investigated. Among the 200 genes included in this gene set, 41 were significantly differentially expressed in HB-279 compared to the other HB models (Supplementary Table 3). Of these 41 genes, six were upregulated and 35 were downregulated in HB-279. The expression of Hallmark Mitotic Spindle genes clearly separated HB-279 from the other HB models (Fig. 9C). Eleven KIF genes are among the Hallmark Mitotic Spindle genes. 7/11 of them had significantly lower expression in HB-279 than in the other HB samples, including *KIF11*.

A correlation analysis with DESeq2 was performed to identify genes whose mRNA expression correlated with the selective drug response rate of filanesib in the in vitro testing in each of the six HB models. No genes significantly correlating with filanesib drug response were identified.

### Filanesib-induced changes on mRNA level

**Fig. 10:**
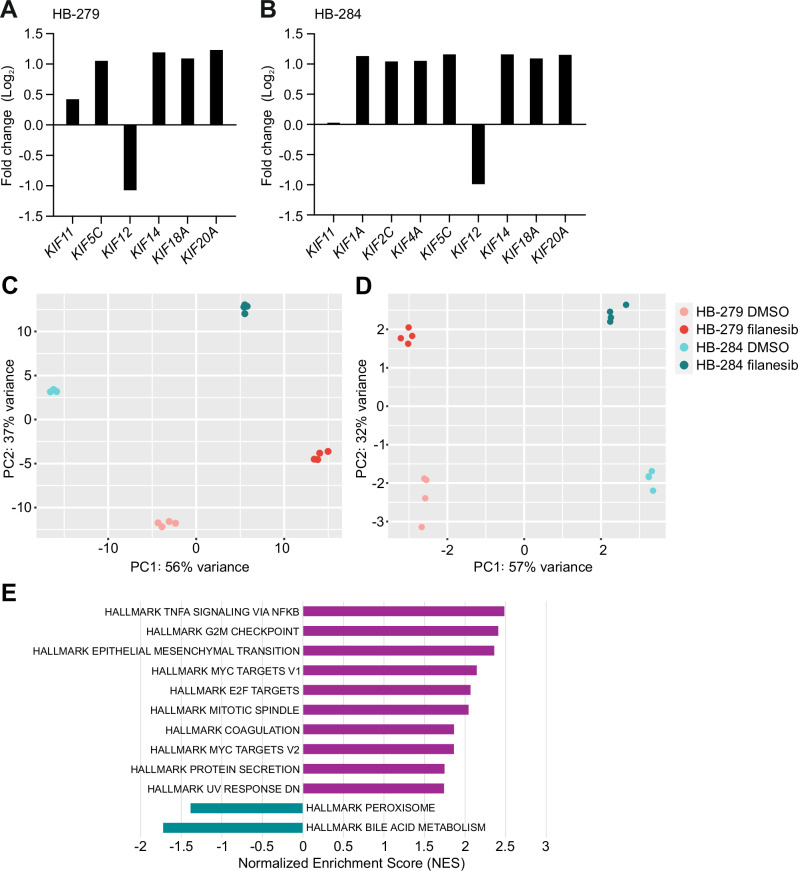
Effects of filanesib-treatment on mRNA-level. Expression of KIF-protein family members after treatment with 10 nM filanesib in HB-279 (**A**) and HB-284 (**B**) cells. Unsupervised principal component analysis of the expression of all genes (**C**) and of a subset of mitotic spindle related genes (**D**) shows the relation between filanesib-treated and DMSO-treated HB-279 and HB-284 models. Gene set enrichment analysis comparing the mRNA expression profiles of filanesib-treated cells (HB-279 and HB-284 combined) compared to untreated cells was done. Top 10 highest and lowest enriched Hallmark gene sets with FDR < 0.25 are shown (**E**). PC principal component, NES normalized enrichment score.

To assess the transcriptomic changes caused by filanesib, RNA sequencing of filanesib and DMSO-treated HB-279 and HB-284 cells was performed. *KIF11* mRNA expression was not significantly affected by filanesib treatment (Fig. 10A, B). However, filanesib treatment increased the expression of other members of the KIF protein family, including *KIF1A*, *KIF2C*, *KIF4A, KIF5C*, *KIF14*, *KIF18A*, and *KIF20A* (Fig. 10A, B). In contrast, *KIF12* expression was downregulated in both HB-279 and HB-284 cells following filanesib treatment (Fig. 10A, B).

An unsupervised PCA of the most variably expressed genes across these two treated and untreated HB models showed that the variance between the treatments (filanesib and DMSO) was greater than the variance between the models (HB-279 and HB-284), with treatment explaining 56% of the variance (PC1) and model 37% (PC2) (Fig. 10C). When analyzing Hallmark Mitotic Spindle genes only, PC1 separated the models and PC2 separated the treatments (Fig. 10D). A paired GSEA analysis was performed to determine the pathways affected by filanesib treatment (Fig. 10E). Filanesib induced overexpression of genes included in Hallmark gene sets for TNFα signaling, G2M Checkpoint, Epithelial-mesenchymal Transition (EMT), and Mitotic Spindle (Fig. 10E).

## Discussion

Few adjustments have been proposed for the pharmaceutical management of HB during the past two decades, and targeted agents have not been incorporated into routine care. Chemotherapeutics utilized in HB treatment predispose the patient to adverse effects, which may be irreversible and affect the health of the individual far into the future. Moreover, patients with high-risk HBs demonstrate a 3-year event-free survival rate below 60%^21^, underscoring the need for better and safer treatment modalities. Herein, we performed an extensive drug screening with 527 compounds using 3D HB cell models established from PDXs with aggressive tumors. Eight of the 527 compounds were found to be effective in all six HB cell models without notable toxicity toward primary pediatric hepatocytes. Two compounds, filanesib and luminespib, were further investigated in this study. Filanesib decreased cell survival in vitro and reduced growth of HB tumors in vivo, suggesting that it could be a potential candidate for future clinical trials.

High-throughput DSRT is utilized to efficiently discover and investigate the effects of multiple compounds simultaneously. Here, we chose to utilize 3D spheroids, which have been shown to better resemble tumor characteristics and behavior in vivo compared to traditional monolayer cultures^22–25^. To the best of our knowledge, this extensive screening has not been performed with 3D HB cell cultures. Over 200 compounds were investigated in a recent study employing patient-derived HB organoids^16^. Similar to our screen, HDAC inhibitors were shown to be effective for multiple HB subtypes^16^. Five of the six HB models used in our study have been investigated earlier in a screen encompassing 60 compounds^17^. There has also been a small screen consisting of 12 drugs using HB organoids^26^. In this screen BET bromodomain inhibitor JQ1 showed some preclinical potential^26^. Our drug library included JQ1; however, it was not among the most effective compounds in our study. Pre-drugged plates were used in our initial drug screen and the results were validated on chosen compounds using readily formed spheroids. The results from these two approaches were consistent as previously demonstrated also by Feodoroff et al.^22^.

In our drug screen, eight of the initial 527 compounds were effective in all six aggressive HB models and showed little to no toxicity toward healthy, age-matched hepatocytes. These eight compounds represented several different mechanisms of action and included an HDAC inhibitor, Hsp inhibitors, an MDM2-antagonist, microtubule inhibitors, and the KSP inhibitor filanesib. Epigenetic modifiers such as HDAC and DNA methyltransferase inhibitors have already previously been under extensive investigation for management of high-risk HBs^16,27,28^. In our study, the HDAC inhibitor fimepinostat (CUDC-907) reduced viability of all HB cell models representing different subtypes. However, we also observed a decrease in the survival of healthy hepatocytes already at concentrations below 100 nM and thus decided to exclude it from further testing.

Three out of eight compounds that were effective in all HB cell models target heat shock protein Hsp90, a molecular chaperone protein that participates in multiple processes in oncogenesis and cancer progression^29^. An elevated Hsp90 expression has been demonstrated in HBs, underlining the reasonableness of this target^30^. One of the three Hsp90 inhibitors, luminespib (also known as AUY922), which has been previously investigated in phase 2 trials for other indications, was chosen for additional analyses^31^. Increased apoptosis was detected in HB spheroids treated with luminespib, but only one model, HB-243, responded slightly to luminespib treatment in vivo. In a previous study conducted using a hepatocellular carcinoma murine model with Huh7 cells, luminespib caused a notable decrease in in vivo tumor growth^32^. Our dosing schedule was less frequent (30 mg/kg of luminespib five times a week for 15 days versus 30 mg/kg three times per week for 18 days), and the route of administration was different from that in the study by Augello et al.^32^ (intraperitoneal injection versus oral gavage administration), which may have affected responses. While results from hepatocellular carcinoma cannot be interpolated directly to HB, more frequent administration should be considered in future HB study designs using Hsp90 inhibitors.

We observed that several mitotic inhibitors decreased the viability of all investigated HB cell models while reducing the survival of primary hepatocytes only at the highest concentrations. Effective drugs included the conventional microtubule inhibitors cabazitaxel and eribulin, as well as the targeted agent filanesib. As of today, the only mitotic inhibitor that has been widely used for HB treatment is vincristine. However, its side effects, such as peripheral neuropathy, limit the use^33,34^. The search for more targeted mitotic inhibitors with fewer side effects has led to the discovery of kinesin spindle protein inhibitors, such as filanesib, litronesib, and ispinesib^35^. Kinesin-like protein 11 (KIF11, also known as kinesin-5 and Eg5) is part of the kinesin superfamily and plays many roles in mitosis, including chromosome positioning, centrosome separation, and the establishment of the bipolar spindle^36–38^. In pediatric cancers, KIF11 has been shown to be overexpressed and to have a prognostic role in Wilms tumors and in acute lymphocytic leukemia^39,40^. Our RNA sequencing data analysis showed overexpression of *KIF11* in all HB PDX cell models. However, there was no correlation between the magnitude of *KIF11* upregulation and response to KIF11 inhibition with filanesib. Thus, predicting who may benefit from KSP inhibitor treatment may be more complex than merely observing the abundance of the target.

It has been shown that inhibition of KIF11 causes mitotic arrest and blocks the cell cycle in metaphase^41^. Consistently, HB cells treated with filanesib in our study were predominantly arrested in the G2/M phase. KSP inhibitors and direct depletion of KIF11 have been demonstrated to cause formation of abnormal monopolar spindles (monoastral phenotype) and mitotic catastrophe, a process leading either to cell death or senescence^42–44^. We observed that the nuclei of HB cells became shattered after filanesib exposure, resembling a typical morphology for mitotic catastrophe.

Filanesib treatment reduced tumor growth in four of five HB PDX models in vivo. These four models predominantly represented the embryonal subtype. Filanesib showed distinctively strong activity in the PDX model HB-279. A remarkable reduction in KIF11 expression was observed in treated HB-279 PDX tissues, confirming that filanesib acts at least partly through KIF11. To explore the mechanisms underlying the strong sensitivity of HB-279 to filanesib, we compared the mRNA expression profiles of all of the HB models and examined the differences in gene expression before and after filanesib treatment. HB-279 clearly differed from the other five HB models by presenting lower expression of many cell cycle related genes and the filanesib-target *KIF11*. Compared to the rest of the models, genes belonging to the Hallmark Mitotic Spindle, Hallmark E2F Targets and Hallmark G2M Checkout sets were mainly downregulated in HB-279 cells compared to the other models. We wonder if the lower baseline expression level of KIF11 and other related genes makes HB-279 more vulnerable to filanesib compared to the rest. The low *KIF11* RNA expression level in HB-279 was reversed when the cells were treated with filanesib, as we observed the upregulation of genes belonging to the aforementioned sets. Interestingly, increased mRNA expression of *KIF*s other than *KIF11* was observed after filanesib treatment, possibly indicating a compensatory mechanism. KIF25 and KIF16A have been hypothesized to function similarly to KIF11, but little is known about their actual functions^45,46^. Further research is required to identify which genes or gene signatures could be employed to identify patients who may benefit from KSP inhibitor treatment.

The only model that showed no response to filanesib in vivo was HB-295 representing fetal HB subtype. However, our in vivo study included only five HB models, and it could be a coincidence that the only PDX with fetal HB histology did not respond to filanesib. The fetal HB models (HB-295, HB-303) had the highest number of hits in our drug screen, suggesting that there are many other compounds to consider for this HB subtype. A more heterogeneous set of HB tumor models is needed for further investigation.

Clinical studies on filanesib have focused on adult multiple myeloma. Currently, there are eight completed phase 1 and phase 2 filanesib trials, and four have published results^47–50^. Adverse events, such as neutropenia, anemia, and fatigue, have mostly been non-cumulative and reversible^51^. There have been no clinical trials with filanesib in HB or other pediatric solid tumor patients. A preclinical neuroblastoma study revealed that filanesib prolongs the survival of orthotopic mice^52^. The only KSP inhibitor that has undergone a phase 1 trial with pediatric solid tumors is ispinesib, which was unfortunately not included in the predefined screening panel used in this study^53^. Our drug screen included three KSP inhibitors: filanesib, SB-743921, and litronesib. SB-743921 has previously been shown to be effective towards pediatric Ewing sarcoma in a preclinical setting^43^. In our screen, SB-743921 showed no toxicity towards healthy controls, but was considered effective in only one of the models, HB-279. To the best of our knowledge, litronesib has not been previously tested in pediatric tumors. In the present study, litronesib demonstrated good efficacy and specificity, with sDSS > 10 in 5/6 of the models. Overall, better understanding of treatment biomarkers and more clinical trials are needed to determine the safety and efficacy of KSP inhibitors in the pediatric population. Based on our findings, we propose that HB should be among the investigated tumor types. Prior to proceeding to phase 1 clinical trials, the potential biomarkers for patient stratification should be further searched, given the variability in treatment responses observed in our study. Clinical trials could then be focused on the HB patient group most likely to benefit from filanesib treatment.

Herein, we investigated 527 compounds using six 3D HB cell models established from PDXs with aggressive tumors, alongside healthy primary cells to assess general toxicity. Our screen revealed eight agents that were effective in all HB models with relatively low toxicity. None of these eight compounds are currently used in the routine care of HB, suggesting that there are novel approaches that may improve patient outcomes. HDACs, proteins mediating mitosis, and Hsp90 were found to be common targets in all HB subtypes. Luminespib was effective in vitro but showed very limited effect in vivo. Filanesib, a KSP inhibitor, efficiently suppressed HB cell viability in vitro and in vivo, particularly in HBs of embryonal origin. Taken together, filanesib is a promising candidate for use in future clinical trials.

## Methods

### Cell models and cell culture

The six HB cell lines established from patient-derived xenografts (PDX; HB-243, HB-279, HB-282, HB-284, HB-295, HB-303) were provided by XenTech (Evry, France)^17^. Human HB cell line HUH6 was obtained from Japanese Collection of Research Bioresources Cell Bank (Osaka, Japan). PDX cells were cultured in Advanced DMEM/F12 (Gibco, Waltham, MA, USA) that was supplemented with 8% fetal bovine serum, 2 nM glutaMAX (Gibco), 100 U/ml penicillin, 100 μg/ml streptomycin sulfate and 20 μM rock kinase inhibitor Y-27632 (Cat#1049; Selleck Chemicals, Houston, TX, USA). HUH6 cells were maintained with Dulbecco’s modified Eagle’s medium (DMEM) supplemented with 10% fetal bovine serum (FBS), 2 nM glutaMAX, 100 U/ml penicillin, and 100 μg/ml streptomycin sulfate (all from Gibco). Primary hepatocytes from 10-year-old white male (HUM191501; Lonza, Basel, Switzerland) were used as controls and cultured as instructed by the manufacturer’s protocol “Guide to spheroid formation using Verified for Spheroids human pediatric hepatocytes”. All cell lines were authenticated through short tandem repeat profiling.

### 3D high-throughput drug sensitivity and resistance testing (DSRT)

Cells were seeded with Biotek MultiFlo FX using a single-channel cassette into 25 µl final volume per well to 384-well U-bottom plates (Corning 3830), pre-drugged using the Echo 550 acoustic dispenser (Labcyte) with a FO5A library of 527 approved and investigational oncology drugs and one combination, each in five concentrations (Supplementary Table 2) [by the High Throughput Biomedicine core unit (FIMM, HiLIFE, University of Helsinki, Finland)]. Cell seeding densities were as follows: HB-303, HB-282, HB-284, and HB-279: 750 cells/well, HB-295 and HB-243: 1000 cells/well. After 72 h incubation, 15 µl CellTiter-Glo 2.0 reagent (CTG, Promega, Madison, WI) was added with Biotek MultiFlo FX. Cell viability (intracellular ATP) was measured using BMG Labtech PHERAstar FS plate reader. Pediatric healthy primary hepatocytes were similarly screened (HUM191501, 500 cells/well, 384-well plate) with FO5A library to evaluate the general toxicity/specificity of the compounds. The quality control for the screens as well as IC50, EC50, and drug sensitivity scores (DSS) were determined for every individual drug from the raw data using FIMM-UH analytics tool Breeze (version 1.0, available https://breeze.fimm.fi/)^19,54^. To assess the robustness of the screen, Z-factors were calculated for the difference in viability at each cell density using dimethyl sulfoxide (DMSO; 0.1%) as a negative control and the antiseptic benzethonium chloride (100 µM) as a positive control. Selective DSS (sDSS) was counted by subtracting the DSS value of similarly screened healthy control hepatocytes from DSS value of cancer cells, method and controls previously described by Chen et al.^55^. Drugs with sDSS ≥ 10 were considered effective.

### Confirmatory viability and apoptosis assays

For validation experiments, 10 mM filanesib (HY-15187) and 10 mM luminespib (HY-10215) in DMSO stocks were obtained from MedChemExpress (New Jersey, USA). Cells were treated with 1, 10, and 100 nM of filanesib or luminespib in all cell experiments if not otherwise described.

HB-243, HB-282, HB-284, HB-279, HB-295, HB-303, and HUH6 cells were seeded at density of 2000 cells/well to low attachment CellCarrier spheroid ULA 96-well-plates (PerkinElmer, Waltham, MA, USA). After 48 h incubation, spheroids were established and treatment with filanesib, luminespib, or control medium with corresponding DMSO concentration was initiated. Viability of spheroids was assessed with ATPlite™ 3D monitoring system (PerkinElmer) as described in the manufacturer’s instructions at treatment timepoint of 72 h. Caspase 3/7 Glo assay (Promega, Madison, WI, USA) was used to measure apoptosis in 3D cultures as instructed. Luminescence was measured with a GloMax microplate reader (Promega, Madison, WI, USA). In HUH6 cells the number of apoptotic cells after *KIF11* silencing was determined by Nicoletti assay^56^.

### Quantitative real-time polymerase chain reaction

Total RNA was extracted from HUH6 cells utilizing NucleoSpin™ RNA, Mini kit (Macherey-Nagel, Düren, Germany) as instructed. Reverse transcription was carried out using iScript cDNA Synthesis Kit (Bio-Rad, Hercules, CA, United States). Quantitative polymerase chain reaction was performed using PowerUp SYBR Green Master Mix (Thermo Fisher Scientific, Fremont, CA, United States). Relative gene expression was assessed using the 2^−ΔΔCT^ method. The geometric mean of *Cyclophilin*, *GAPDH*, and *PPIB* served as a reference. Primer sequenced were designed as follows: *Cyclophilin* 5′ -CAATGGCCAACAGAGGGAAG—3′ (forward), 5′ -CCAAAAACAACATGATGCCC—3′ (reverse); *GAPDH* 5′ -GGTCATCCATGACAACTTTGG—3′ (forward), 5′ -CCATCCACAGTCTTCTGGGT—3′ (reverse); *PPIB* 5′ -CAATGGCCAACAGAGGGAAG—3′(forward), 5′—CCAAAAACATGATGCCCA—3′ (reverse); *KIF11* 5′ -GATGGACGTAAGGCAGCTCA —3′ (forward), 5′ -TGTGGTGTCGTACCTGTTGG—3′ (reverse).

### Protein extraction and western blotting

Proteins were extracted utilizing ProteoSpin Detergent-free total protein isolation kit (Norgen Biotek Corp., Thorold, Canada). Fifteen micrograms of protein was separated by electrophoresis using Mini-Protean TGX Stain-Free Gels (Bio-Rad). Proteins were transferred onto polyvinyl fluoride membrane and non-specific binding was blocked with 5% non-fat milk in 0.1% Tris-buffered Tween saline buffer. Membranes were incubated with the following primary antibodies at +4 °C for overnight: anti-human EG5 rabbit mAb in dilution 1:750 (#14404S, Cell Signaling Technology, Danvers, MA, USA). Next, goat anti-rabbit IgG secondary antibody (#111-035-144 in dilution 1:10,000; Jackson ImmunoResearch, West Grove, PA, USA) incubation was performed at room temperature for 1 h. Protein bands were illuminated utilizing the Enhanced Chemiluminescence detection kit (Amersham ECL reagent; GE Healthcare, Barrington, IL) and analyzed with Image Lab Software 6.0 (Bio-rad). KIF11 band intensities were normalized to amount of total protein in corresponding lane (Supplementary Fig 7) utilizing stain-free technology^57^.

### Cell phenotype analysis and imaging

HB cell lines HB-243, HB-279, HB-282, and HB-284 were cultured and treated with filanesib or luminespib as described above. Cells were seeded on 96-well PhenoVue plates (PerkinElmer, Waltham, MA, USA); 6000–13,000 cells per well, and after 24 h treated with filanesib or luminespib for another 24 h. DMSO 0,1% was used as control. PhenoVue Cell Painting JUMP kit (PerkinElmer) was used to stain the cells according to the Cell Painting protocol with some modifications (JUMP protocol v2)^58^. In brief, two separate staining solutions with mitochondrial stain, wheat germ agglutin (WGA), concavalin A, Hoechst and nuclear acid stain were prepared. Cells were first stained with mitochondrial stain and incubated at 37 °C, after which they were fixed with methanol-free PFA, washed with Hank’s balanced salt solution (HBSS) and then stained with the rest of the stains.

Cells were then imaged with a high content confocal microscope (PerkinElmer Opera Phenix; FIMM High Content Imaging and Analysis Unit FIMM-HCA, FIMM, HiLIFE, UH, Finland) using a 40x water immersion objective (NA 1.1) and 7 optical Z-planes with 1 µm interval to optimize the spatial resolution of features in filanesib-treated cells. In this system, we used the confocal mode for imaging which utilizes four separate channels. The used labels and wavelengths were Hoechst 33342 for DNA (405 nm ex/435–480 nm em); Concavalin A/Alexa Fluor 488 for endoplasmic reticulum (488 ex/500–550 em); PhenoVue Fluor 568 phalloidin, PhenoVue Fluor 555—WGA and PhenoVue 512 Nuclear Acid stain conjugate (561 ex/570–630 em) and PhenoVue 641 for mitochondria (640 ex/650–760 em).

### High-content image analysis

The images obtained from Cell Painting assay by high-content microscopic imaging were analyzed in single-cell level using CellProfiler 3.1.8^59^. The Hoechst channel was first converted to binary image using minimum cross entropy (MCE) based automated thresholding. Individual nuclei were labeled using marker-based watershed transform from the seed points of local maxima of the distance transformed binary image. The outcome label image was masked with the thresholded Hoechst channel to get the final segmented nuclei objects. All nuclei with a diameter of less than 40px or more than 240px were discarded. The cells were segmented by propagating from the segmented nuclei objects to MCE thresholded Alexa488 (endoplasmic reticulum) channel. In total, 300 intensity, morphological and texture features were measured from each segmented cell for downstream analysis.

Jupyter Notebook using Python 3.6.13 was developed for downstream analysis. As a preprocessing step, all features were normalized plate-wise using standard scaling of the negative control samples (DMSO). The scaled single-cell features were visualized separately for each PDX model using Uniform Manifold Approximation and Projection (UMAP)^60^. The similarity of treatment responses was studied using data of all PDX models by hierarchical clustering with Euclidean distance metric.

### Cell cycle analysis

HB-279, HB-284, HB-243, and HUH6 cells (5.0 × 10^5^) were seeded in six-well plates and allowed to adhere for 24 h. Next, HB-279, HB-284, and HB-243 cells were treated with 10 nM filanesib or 50 nM luminespib or DMSO for 24 h (filanesib) or 48 h (luminespib), trypsinized, and fixed with 70% ethanol at −4 °C. HUH6 cells were transfected with NT siRNA or *KIF11* siRNA for 72 h, trypsinized, and fixed with 70% ethanol at −4 °C. Fixed cells were washed with 1% BSA in PBS, followed by incubation with FxCycle PI/RNase Staining Solution for 30 min at RT. Cell cycle analysis was conducted using Novocyte Quanteon (Agilent, CA, USA), flow cytometer, and the results were analyzed with NovoExpress software (Agilent). The flow cytometry analysis was performed at the HiLife Flow Cytometry Unit, University of Helsinki.

### *KIF11* silencing

*KIF11* expression was inhibited in HUH6 cells via small interfering RNA (siRNA) transfection. Briefly, adherent HUH6 cells were exposed to 25 nM of *KIF11* ON-TARGETplus SMARTpool siRNA or ON-TARGETplus non-targeting (NT) control siRNA (both from Horizon Discovery, Cambridge, UK). Dharmafect 1 (Horizon Discovery) was used to deliver siRNAs into the HUH6 cells. Knockdown efficacy was evaluated at mRNA and protein level 72 h after initiation of transfection. Cells were transfected following manufacturer’s instructions.

### In vivo experiments

Each study arm consisted of three mice per PDX. Five different PDXs in total were used. Thus, filanesib arm consisted of 15 mice, luminespib arm had 15 mice and the control group 15 mice. 20 mm3 tumor pieces of HB-243, HB-279, HB-282, HB-284, and HB-295 were subcutaneously implanted into the interscapular area of each female athymic nude mouse (Athymic Nude-Foxn1 nu, ENVIGO, Gannat, France). Mice that met the inclusion criteria of 60–200 mm^3^ tumors were then after the latency period randomly assigned to one of the study arms. Drugs were administered via oral gavage. Dose of filanesib was 20 mg/kg every 4th day and for luminespib 30 mg/kg three times per week. The treatment period was 18 days or shorter if sacrification criteria was met earlier. A mixture of 10% DMSO, 40% PEG300, 5% tween 80, 45% NaCl 0.9% was used as the vehicle. Animals were weighted at tumor measurement times and monitored daily for physical changes, behavior, and clinical observations. Tumor volume (TV) was evaluated by measuring diameters with a caliper 2–3 times a week using the formula TV (mm^3^) = [length (mm) × width (mm)^2^]/2 (length and width being the longest and the shortest diameters of the tumor). The antitumoral effect was assessed by the ratio of mean tumor volume in treated versus control groups. Animals were euthanized by cervical dislocation or by CO_2_ inhalation followed by observation of rigor mortis. No analgesic drug was used for pain. All experiments were performed in accordance with French legislation concerning the protection of laboratory animals and in accordance with a currently valid license for experiments on vertebrate animals, issued by the French Ministry of Higher Education, Research and Innovation (APAFIS#27068-2017090717367058 v2).

Fresh tumor samples were collected from all mice for FFPE at the end of the study or at the group endpoint, without specific delay after the last dosing. The tumor was fixed in 10% formalin for 24 h, transferred in 70% ethanol for maximum 2 weeks and transferred to further sampling for immunohistochemistry

### Immunohistochemistry

Murine HB tumor sections were stained as follows: samples were deparaffinized and 10 mM Tris-1mM EDTA pH9, 20 at min +99 °C was used for antigen target retrieval. 0.9% H2O2 in TBS, 15 min RT was used to block endogenous peroxidase activity. Nonspecific binding was blocked using TBST/ 10% Normal Goat Serum, 15 min RT. Following primary antibodies were used: Rabbit anti KIF11 (PA5-82394, Invitrogen, dilution 1:300) and rabbit anti KIF-15 (PA5-57305, Invitrogen, dilution 1:150) for 1 h RT. Secondary antibody was BrightVision (DPVR55HRP, ImmunoLogic). BrightDAB (BS04-110, ImmunoLogic) was used to visualize the bound antibody.

Images were generated using 3DHISTECH Pannoramic 250 FLASH II digital slide scanner at the FIMM Digital Microscopy and Molecular Pathology Unit (Helsinki Institute of Life Science-HiLIFE, University of Helsinki, and Biocenter Finland).

### RNA sequencing and analysis

RNA sequencing protocols for all six untreated HB PDX models are previously described by Kats et al.^17^. Raw RNA sequencing datasets from previously published studies were obtained from European Genome-phenome Archive (EGA) (https://ega-archive.org/). Accession number for untreated HB-282, HB-295, HB-279, HB-284 and HB-243 cells is EGAS00001004827/EGAD00001006621 and for HB-303 EGAS50000000928/EGAD5000000135. Comparison of untreated HB cell lines to normal hepatocytes has been previously described by Nousiainen et al.^61^.

Cell models HB-279 and HB-284 were cultured and treated with 10 nM filanesib or DMSO as described above. Total protein and RNA were extracted after 24 h incubation using NucleoSpin RNA/Protein extraction kit (Macherey-Nagel, Düren, Germany). Instructions provided by the manufacturer were followed. Sequencing of filanesib-treated HB-279 and HB-282 cells was completed by Genewiz, Leipzig, Germany. Samples went through quality control by fragment analysis before sequencing. RNA libraries were prepared applying polyA selection, after which Illumina compatible cDNA libraries were constructed. Raw RNA sequencing datasets have been deposited to EGA, accession number EGAS50000000899/EGAD50000001314.

The online tool Chipster was used for the analysis of the RNA sequencing data (version 4.0, available https://chipster.rahtiapp.fi/)^62^. The tool FastQC was used for quality control. Adapters were trimmed using Trimmomatic. Reads were then mapped to human reference genome Homo_sapiens GRCh38.95 using HISAT2 for paired end reads^63^. Aligned reads per genes were counted using HTseq^64^. Differential gene expression analyses were performed using DESeq2 (R 4.2.1, DESeq2 1.38.3). Genes were considered significantly differentially expressed with an adjusted p ≤ 0.1 for a minimum absolute log2 fold change of 0.6. Gene set enrichment analysis was performed with GSEA (4.3.2; preranked on the DESeq2 log2FC estimate and using the weighted scoring scheme). FDR < 0.25 was considered significant.

### Statistical analyses

For viability assays, apoptosis measurements, and cell cycle analysis, statistical significance was assessed using Student’s *t*-test. *p < 0.05 was considered as a statistically significant and **p < 0.01 as a statistically highly significant. All experiments were performed at least in triplicates.

## Supplementary information

Supplemental Material

## Data Availability

The RNA sequencing datasets generated and/or analyzed during the current study are publicly available in the European Genome-phenome Archive (EGA) (https://ega-archive.org/, accession number EGAS00001004827/EGAD00001006621 for untreated HB-282, HB-295, HB-279, HB-284, and HB-243, accession number EGAS50000000928/EGAD5000000135 for untreated HB-303 and accession number EGAS50000000899/EGAD50000001314 for filanesib-treated HB-279 and HB-284). Other data, such as detailed drug screen results, can be provided by the corresponding author upon justified request.

## Code availability

Publicly available codes have been cited in the related Methods sections. Scripts used in the high-content image analysis are available via GitHub repository (https://github.com/lopaavol/filanesib-hepatoblastoma).
