# Supplementary material for: Functional screening identifies kinesin spindle protein inhibitor filanesib as a potential treatment option for hepatoblastoma
Source: NPJ Precis Oncol. 2025 Apr 25;9:122. doi: 10.1038/s41698-025-00915-8 (PMC12032252; doi:10.1038/s41698-025-00915-8)
Supplement: Supplementary file 1 — Supplemental Material [file 41698_2025_915_MOESM1_ESM.pdf]

Supplementary Material

Functional screening identifies kinesin spindle protein inhibitor filanesib as a potential treatment option for hepatoblastoma

Ruth Nousiainen, Katja Eloranta, Jani Saarela, Antti Hassinen, Tamara J Luck, Stefano Cairo, Emilie Indersie, Swapnil Potdar, Michaela J Feodoroff, Jouko Lohi, Lassi Paavolainen, David B Wilson, Vilja Pietiäinen, Markku Heikinheimo, Marjut Pihlajoki

Table of Contents

Supplementary Table 1……………………………………………………………………… 2
Supplementary Table 2……………………………………………………………………… 3
Supplementary Figure 1…………………………………………………………………….. 4
Supplementary Figure 2…………………………………………………………………….. 5
Supplementary Figure 3…………………………………………………………………….. 6
Supplementary Figure 4…………………………………………………………………….. 7
Supplementary Figure 5…………………………………………………………………….. 9
Supplementary Figure 6…………………………………………………………………… 10
Supplementary Table 3…………………………………………………………………….. 11
Supplementary Figure 7…………………………………………………………………… 12

Supplementary Table 1.

Demographical and clinical information of the HB models. PDX were derived from five HB patients with six HB tumors.

Sample ID	HB-243	HB-279	HB-282	HB-284	HB-295	HB-303
**Age at sampling (months)**	52	79	12	83	26	69
**Sex**	male	male	male	male	female	female
**Sample type**	intrahepatic relapse	primary tumor	primary tumor	peritoneal metastasis of HB-279	primary tumor	primary tumor
**Main histological component**	embryonal	embryonal & macrotrabecular	embryonal	embryonal	fetal	fetal
**Beta-catenin mutation status**	exon 3 deletion	deletion 24aa exon 3	G34R	deletion 24aa exon 3	exon 3 deletion	wild type
**Metastasis**	no	yes (HB-284)	no	n/a	yes	no
**PRETEXT**	n/a	IV	II	n/a	II	II
**Chemo protocol**	carboplatin + etoposide	SIOPEL-4	SIOPEL6+3	etoposide + cisplatin	SIOPEL-4	cisplatin 4 cycles
**AFP serum at diagnosis (ng/mL)**	6 000	1 000 000	1 286 000	2 162	585 350	158 645
**AFP serum post-chemo (ng/mL)**	5 000	30 000	1 000 000	1 089	1 400	26 000

Supplementary Table 2.

List of the 527 compounds and one drug combination used in the DSRT. Compounds are listed in alphabetical order.

List of tested compounds

1-methyl-D-tryptophan	BCI	Digoxin	GSK923295	Neflamapimod	Quizartinib	Temozolomide
4-hydroxytamoxifen	Belinostat	Dinaciclib	GSK-J4	Nelarabine	Rabusertib	Temsirolimus
8-amino-adenosine	Bentamapimod	Disulfiram(+CuCl2)	Hydroxyfasudil	Neratinib	Radotinib	Teniposide
8-chloro-adenosine	Bexarotene	Docetaxel	Hydroxyurea	Nilotinib	Ralimetinib	Tepotinib
A-1155463	BGB-283	Doramapimod	I-BET151	Nilutamide	Raloxifene	Tesevatinib
A-1210477	BGB324	Dovitinib	Ibrutinib	Nintedanib	Raltitrexed	TEW-7197
A-1331852	BI 2536	Doxorubicin	Icotinib	Niraparib	Ravoxertinib	TG100-115
A-366	Bicalutamide	Duvelisib	Idarubicin	NMS-873	Regorafenib	TGR-1202
A-419259	BIIB021	E7820	Idasanutlin	NVP-AEW541	Resatorvid	TGX-221
ABC294640	Bimatoprost	Eltanexor	Idelalisib	NVP-BGT226	Resiquimod	TH588
Abemaciclib	Binimetinib	Enasidenib	Imatinib	NVP-BHG712	Resminostat	Thalidomide
Abexinostat	Birabresib	Encorafenib	Imiquimod	NVP-CGM097	RGFP966	Thioguanine
Abiraterone	Birinapant	ENMD-2076	Indibulin	NVP-LCL161	Ribociclib	THZ2
ABT-751	Bleomycin	Ensartinib	Infigratinib	NVP-LGK974	Ridaforolimus	TIC10
Acalabrutinib	BMS-754807	Entinostat	IOX-1	NVP-RAF265	Rigosertib	Tideglusib
Acitretin	BMS-777607	Entospletinib	IOX-2	NVP-SHP099	Ripasudil	Tipifarnib
Afatinib	BMS863233	Entrectinib	Ipatasertib	ODM-201	RO5126766	Tirabrutinib
Afuresertib	BMS-911543	Enzalutamide	Itraconazole	Olaparib	Rociletinib	Tivantinib
Aldoxorubicin	Bortezomib	Enzastaurin	Ivosidenib	Olmutinib	Rocilinostat	Tivozanib
Alectinib	Bosutinib	Epacadostat	Ixabepilone	Omacetaxine	Romidepsin	Tofacitinib
Alisertib	BRD7116	Epirubicin	Ixazomib	Omaveloxolone	Roxadustat	Topotecan
Allopurinol	Brigatinib	EPZ015666	JPH203	Omipalisib	RSL3	Toremifene
Alpelisib	Brivanib	EPZ031686	JQ1	Onalespib	Ruboxistaurin	Tosedostat
Altiratinib	Bryostatin 1	EPZ-5687	KD025	ONX-0914	Rucaparib	Tozasertib
Alvocidib	Buparlisib	Erastin	KU-60019	Oprozomib	Ruxolitinib	TRAM-34
Amcasertib	BX-912	Erdafitinib	Lapatinib	Orteronel	S-63845	Trametinib
AMG-232	C646	Eribulin	Larotrectinib	Osimertinib	Sabutoclax	Tretinoin
AMG319	Cabazitaxel	Erlotinib	Lasofoxifene	OSU-03012	Salinomycin	Triapine
AMG-337	Cabozantinib	Etoposide	Lenalidomide	OTS167	Sapanisertib	Triciribine
AMG-925	Canertinib	Everolimus	Lenvatinib	OTS-964	Sapitinib	Trifluridine
Aminoglutethimide	Capecitabine	Exemestane	Letrozole	Oxaliplatin	SAR405838	Tubacin
Amsacrine	Capmatinib	Fedratinib	Linifanib	PAC-1	Saracatinib	Tubastatin A
Amuvatinib	Carboplatin	Filanesib	Linsitinib	Paclitaxel	Saridegib	Tucatinib
Anagrelide	Carfilzomib	Filgotinib	Litronesib	Pacritinib	SB 743921	Tucidinostat
Anastrozole	CC-115	Finasteride	Lomeguatrib	Palbociclib	SCH772984	UCN-01
Apalutamide	CC122	Fingolimod	Lonafarnib	Palomid-529	Seliciclib	Ulixertinib
Apatinib	CC-223	Floxuridine	Losmapimod	Panobinostat	Selinexor	UM729
APR-246	CCT196969	Fludarabine	Lovastatin	Pazopanib	Selonsertib	UNC0638
AR-42	Cediranib	Fluorouracil	Lucitanib	PCI-34051	Selumetinib	UNC0642
Arsenic(III) oxide	Celecoxib	Flutamide	Luminespib	PD0325901	Senexin B	UNC1215
ARV-825	CEP-32496	Foretinib	LY-2584702	Peficitinib	Sepantronium bromide	UNC2881
Asciminib	CEP-37440	Fostamatinib	LY-2874455	Pemetrexed	Serabelisib	Upadacitinib
ASP3026	Cerdulatinib	FRAX486	LY3009120	Pentostatin	SGC0946	Uprosertib
AT 101	Ceritinib	Fulvestrant	LY3023414	Perifosine	SGC-CBP30	URB597
AT13148	Chloroquine	Galiellalactone	Marimastat	Pevonedistat	SGI-1776	Valproic acid
AT-406	Cilengitide	Galunisertib	Masitinib	Pexidartinib	SH-4-54	Valrubicin
AT7519	Cisplatin	Gandotinib	Megestrol acetate	PF-00477736	Silmitasertib	Vandetanib
AT9283	Cladribine	Ganetespib	Mepacrine	PF-00562271	Simvastatin	Varespladib
Atorvastatin	Clofarabine	GDC-0084	Mercaptopurine	PF-03758309	Sirolimus	Varlitinib
Auranofin	Clomifene	GDC-0623	Merestinib	PF-04708671	Sitravatinib	Vatalanib
AVN944	Cobimetinib	GDC-0853	Metformin	PF-06463922	SN-38	VE-821
Axitinib	Copanlisib	GDC-0919	Methotrexate	PF06650833	SNS-032	Veliparib
AZ 3146	CPI-0610	Gedatolisib	Methylprednisolone	PF-3845	Sonidegib	Vemurafenib
AZ191	CPI-360	Gefitinib	Midostaurin	PF-4800567	Sonolisib	Venetoclax
Azacitidine	CPI-613	Gemcitabine	Milciclib	PF-670462	Sorafenib	VER 155008
AZD0156	Crenolanib	Gilteritinib	Miltefosine	PFI-1	Sotrastaurin	Verdinexor
AZD-1080	Crizotinib	Givinostat	Mitomycin C	PH-797804	Spebrutinib	Vesatolimod
AZD1152-HQPA	CUDC-305	Glasdegib	Mitotane	PHA 408	StemRegenin 1	VGX-1027
AZD1208	CUDC-907	Glesatinib	Mitoxantrone	Pictilisib	Sunitinib	Vidofludimus
AZD1480	Cytarabine	GNE-0877	Mivebresib	Pilocarpine	Tacedinaline	Vinblastine
AZD1775	Cytarabine/Idarubicin	GNE-7915	MK-0752	PIM-447	Tacrolimus	Vincristine
AZD3759	Dabrafenib	Golvatinib	MK-2206	Pinometostat	TAK-285	Vinflunine
AZD3965	Dacomitinib	Goserelin	MK-8745	Pirfenidone	TAK-530	Vinorelbine
AZD4547	Dactinomycin	GSK-1070916	MK-8776	Pixantrone	TAK-901	Vismodegib
AZD-5363	Dactolisib	GSK2256098	ML323	Plerixafor	Taladegib	Vistusertib
AZD-5438	Danusertib	GSK-2334470	ML390	Plicamycin	Talazoparib	VLX1570
AZD-6482	Daporinad	GSK2636771	Mocetinostat	Pomalidomide	Talmapimod	Volasertib
AZD6738	Darapladib	GSK2656157	Molibresib	Ponatinib	Tamatinib	Vorinostat
AZD7545	Dasatinib	GSK269962	Momelotinib	Poziotinib	Tamoxifen	VS-4718
AZD7762	Daunorubicin	GSK2801	Motesanib	Pracinostat	Tandutinib	WEHI-539
AZD8055	dBET1	GSK2830371	Motolimod	Pravastatin	Tanzisertib	XAV-939
AZD-8186	Decernotinib	GSK2879552	MST-312	Prednisolone	Tarenflurbil	ZSTK474
Bafetinib	Decitabine	GSK343	Mubritinib	Prexasertib	Taselisib	
Baricitinib	Deferoxamine	GSK-461364	Napabucasin	PS-1145	Tasquinimod	
BAY 87-2243	DEL-22379	GSK650394	Navitoclax	PTC-209	Tazemetostat	
BAY-1436032	Dexamethasone	GSK-690693	Necrostatin 2	Quisinostat	Telatinib	

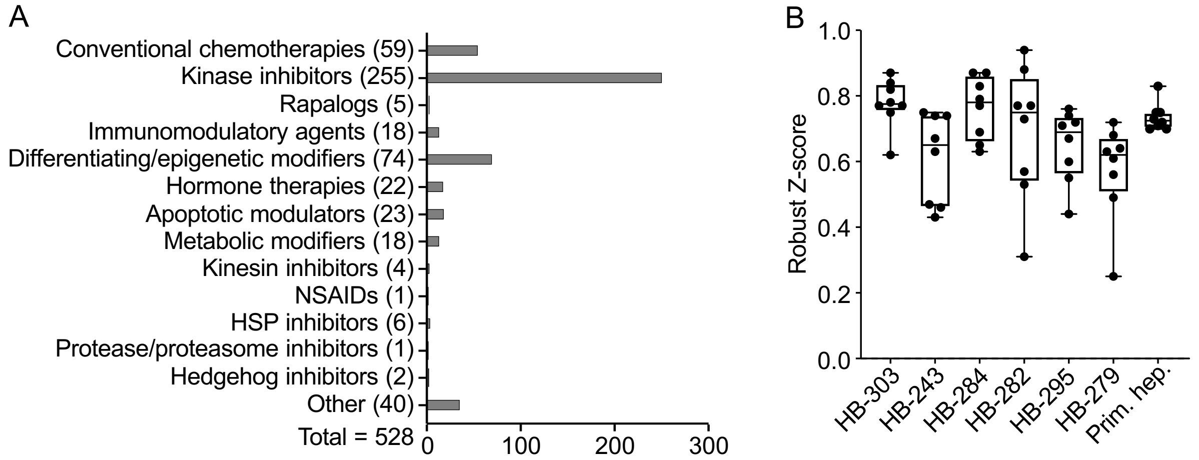

Supplementary Figure 1. Technical overview of the high-throughput drug screen. The number of screened compounds listed by the mechanism of action (A). Z-scores were calculated to assess the robustness of the drug screen (B).

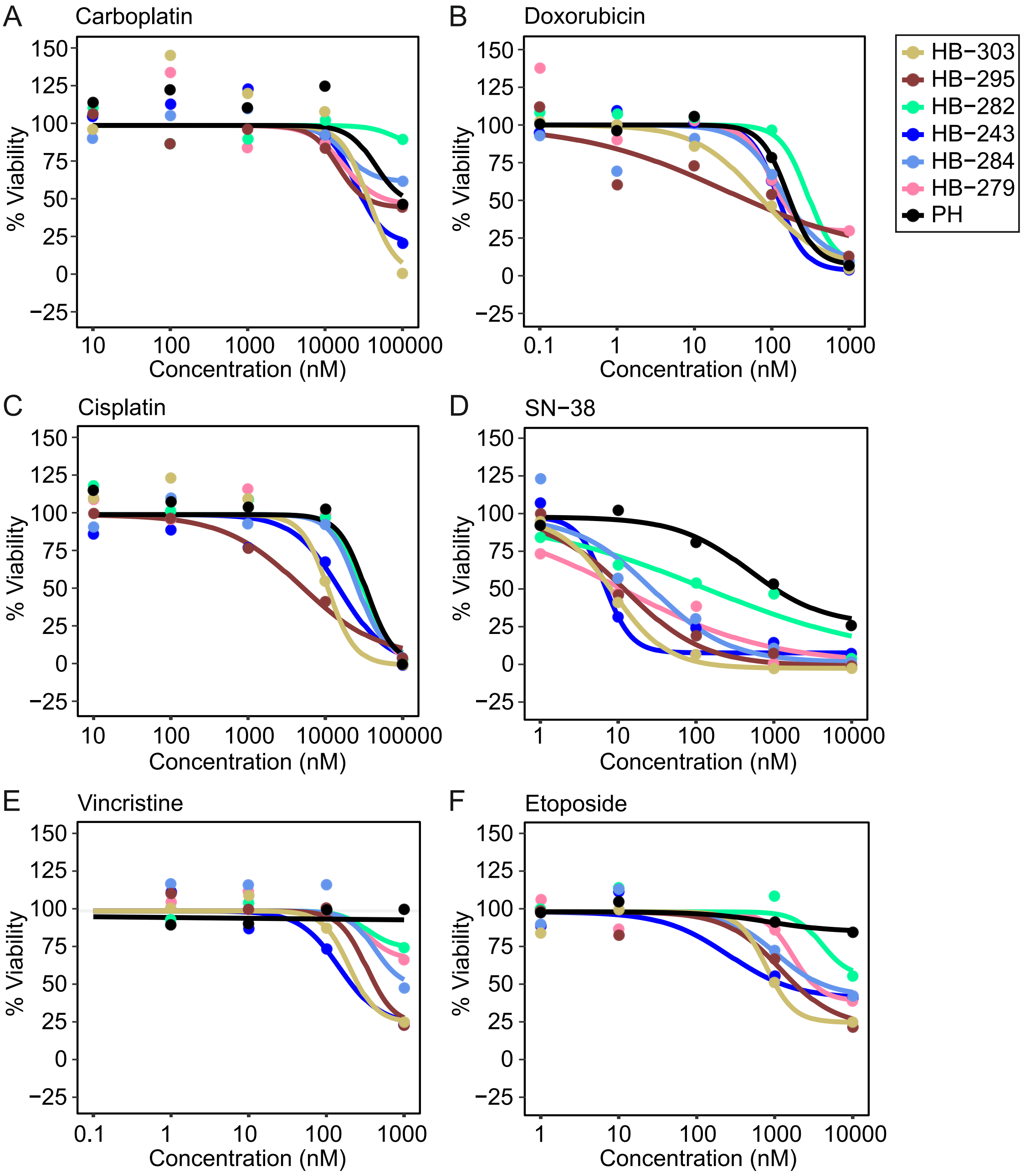

Supplementary Figure 2. Dose-response curves of conventional HB chemotherapeutics. Carboplatin (A), doxorubicin (B), cisplatin (C), vincristine (E), and etoposide (F) were effective only with the highest tested doses in most of the models. HB PDX cell models were responsive to SN–38, the active metabolite of irinotecan (D). PH = primary hepatocytes.

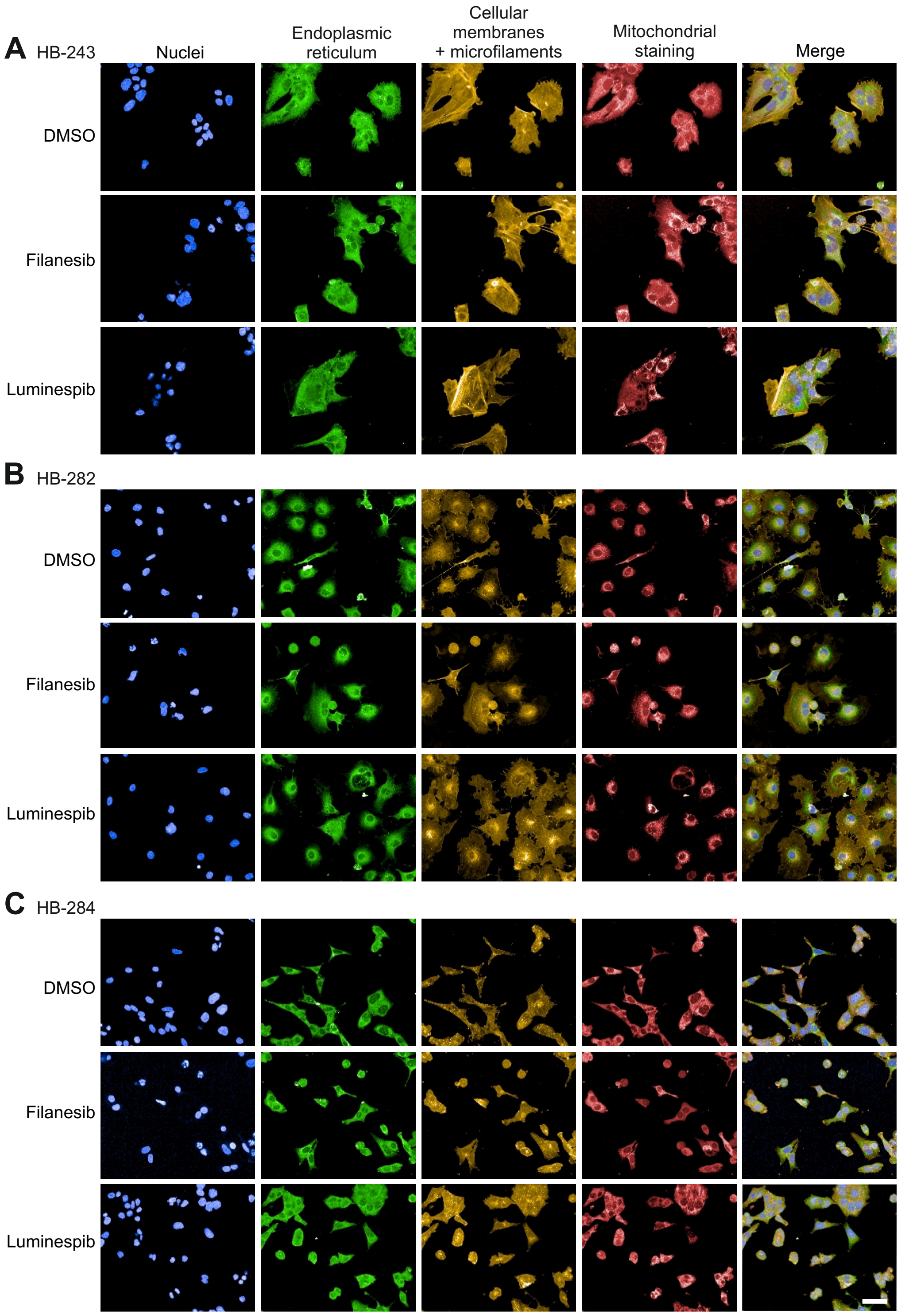

Supplementary Figure 3. Images of cellular morphological features from cell painting. HB cell models HB-243 (A), HB-282 (B) and HB-284 (C). Four different wave lengths were used to measure the morphological features. Different stains were nuclear stain, endoplasmic reticulum, cellular membranes and microfilaments, and mitochondria. Scale bar = 50 µm.

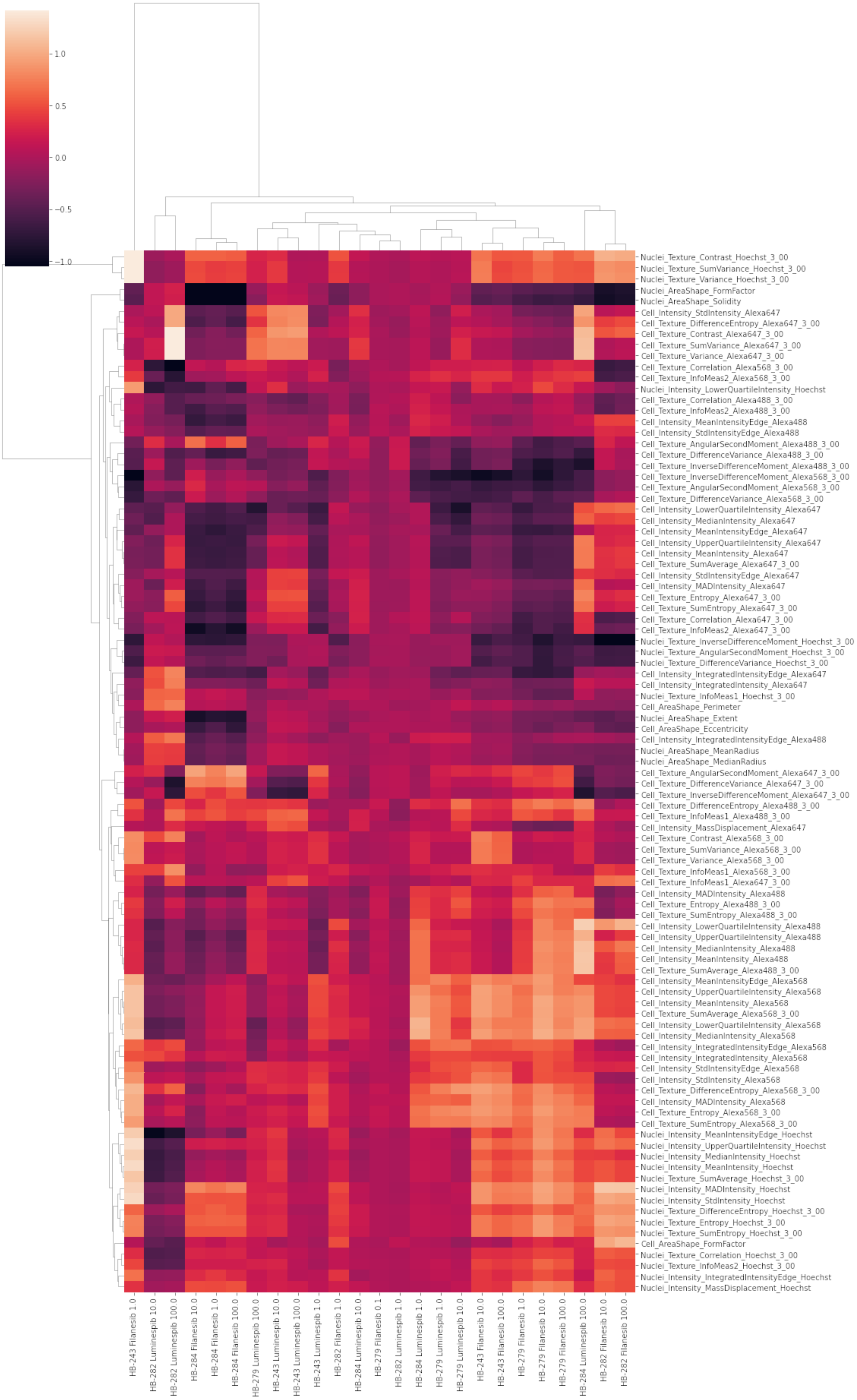
1.0
0.5
0.0
−0.5
−1.0
Nuclei_Texture_Contrast_Hoechst_3_00
Nuclei_Texture_SumVariance_Hoechst_3_00
Nuclei_Texture_Variance_Hoechst_3_00
Nuclei_AreaShape_FormFactor
Nuclei_AreaShape_Solidity
Cell_Intensity_StdIntensity_Alexa647
Cell_Texture_DifferenceEntropy_Alexa647_3_00
Cell_Texture_Contrast_Alexa647_3_00
Cell_Texture_SumVariance_Alexa647_3_00
Cell_Texture_Variance_Alexa647_3_00
Cell_Texture_Correlation_Alexa568_3_00
Cell_Texture_InfoMeas2_Alexa568_3_00
Nuclei_Intensity_LowerQuartileIntensity_Hoechst
Cell_Texture_Correlation_Alexa488_3_00
Cell_Texture_InfoMeas2_Alexa488_3_00
Cell_Intensity_MeanIntensityEdge_Alexa488
Cell_Intensity_StdIntensityEdge_Alexa488
Cell_Texture_AngularSecondMoment_Alexa488_3_00
Cell_Texture_DifferenceVariance_Alexa488_3_00
Cell_Texture_InverseDifferenceMoment_Alexa488_3_00
Cell_Texture_InverseDifferenceMoment_Alexa568_3_00
Cell_Texture_AngularSecondMoment_Alexa568_3_00
Cell_Texture_DifferenceVariance_Alexa568_3_00
Cell_Intensity_LowerQuartileIntensity_Alexa647
Cell_Intensity_MedianIntensity_Alexa647
Cell_Intensity_MeanIntensityEdge_Alexa647
Cell_Intensity_UpperQuartileIntensity_Alexa647
Cell_Intensity_MeanIntensity_Alexa647
Cell_Texture_SumAverage_Alexa647_3_00
Cell_Intensity_StdIntensityEdge_Alexa647
Cell_Intensity_MADIntensity_Alexa647
Cell_Texture_Entropy_Alexa647_3_00
Cell_Texture_SumEntropy_Alexa647_3_00
Cell_Texture_Correlation_Alexa647_3_00
Cell_Texture_InfoMeas2_Alexa647_3_00
Nuclei_Texture_InverseDifferenceMoment_Hoechst_3_00
Nuclei_Texture_AngularSecondMoment_Hoechst_3_00
Nuclei_Texture_DifferenceVariance_Hoechst_3_00
Cell_Intensity_IntegratedIntensityEdge_Alexa647
Cell_Intensity_IntegratedIntensity_Alexa647
Nuclei_Texture_InfoMeas1_Hoechst_3_00
Cell_AreaShape_Perimeter
Nuclei_AreaShape_Extent
Cell_AreaShape_Eccentricity
Cell_Intensity_IntegratedIntensityEdge_Alexa488
Nuclei_AreaShape_MeanRadius
Nuclei_AreaShape_MedianRadius
Cell_Texture_AngularSecondMoment_Alexa647_3_00
Cell_Texture_DifferenceVariance_Alexa647_3_00
Cell_Texture_InverseDifferenceMoment_Alexa647_3_00
Cell_Texture_DifferenceEntropy_Alexa488_3_00
Cell_Texture_InfoMeas1_Alexa488_3_00
Cell_Intensity_MassDisplacement_Alexa647
Cell_Texture_Contrast_Alexa568_3_00
Cell_Texture_SumVariance_Alexa568_3_00
Cell_Texture_Variance_Alexa568_3_00
Cell_Texture_InfoMeas1_Alexa568_3_00
Cell_Texture_InfoMeas1_Alexa647_3_00
Cell_Intensity_MADIntensity_Alexa488
Cell_Texture_Entropy_Alexa488_3_00
Cell_Texture_SumEntropy_Alexa488_3_00
Cell_Intensity_LowerQuartileIntensity_Alexa488
Cell_Intensity_UpperQuartileIntensity_Alexa488
Cell_Intensity_MedianIntensity_Alexa488
Cell_Intensity_MeanIntensity_Alexa488
Cell_Texture_SumAverage_Alexa488_3_00
Cell_Intensity_MeanIntensityEdge_Alexa568
Cell_Intensity_UpperQuartileIntensity_Alexa568
Cell_Intensity_MeanIntensity_Alexa568
Cell_Texture_SumAverage_Alexa568_3_00
Cell_Intensity_LowerQuartileIntensity_Alexa568
Cell_Intensity_MedianIntensity_Alexa568
Cell_Intensity_IntegratedIntensityEdge_Alexa568
Cell_Intensity_IntegratedIntensity_Alexa568
Cell_Intensity_StdIntensityEdge_Alexa568
Cell_Intensity_StdIntensity_Alexa568
Cell_Texture_DifferenceEntropy_Alexa568_3_00
Cell_Intensity_MADIntensity_Alexa568
Cell_Texture_Entropy_Alexa568_3_00
Cell_Texture_SumEntropy_Alexa568_3_00
Nuclei_Intensity_MeanIntensityEdge_Hoechst
Nuclei_Intensity_UpperQuartileIntensity_Hoechst
Nuclei_Intensity_MedianIntensity_Hoechst
Nuclei_Intensity_MeanIntensity_Hoechst
Nuclei_Texture_SumAverage_Hoechst_3_00
Nuclei_Intensity_MADIntensity_Hoechst
Nuclei_Intensity_StdIntensity_Hoechst
Nuclei_Texture_DifferenceEntropy_Hoechst_3_00
Nuclei_Texture_Entropy_Hoechst_3_00
Nuclei_Texture_SumEntropy_Hoechst_3_00
Cell_AreaShape_FormFactor
Nuclei_Texture_Correlation_Hoechst_3_00
Nuclei_Texture_InfoMeas2_Hoechst_3_00
Nuclei_Intensity_IntegratedIntensityEdge_Hoechst
Nuclei_Intensity_MassDisplacement_Hoechst
HB-243 Filanesib 1.0
HB-282 Luminespib 10.0
HB-282 Luminespib 100.0
HB-284 Filanesib 10.0
HB-284 Filanesib 1.0
HB-284 Filanesib 100.0
HB-279 Luminespib 100.0
HB-243 Luminespib 10.0
HB-243 Luminespib 100.0
HB-243 Luminespib 1.0
HB-282 Filanesib 1.0
HB-284 Luminespib 10.0
HB-279 Filanesib 0.1
HB-282 Luminespib 1.0
HB-284 Luminespib 1.0
HB-279 Luminespib 1.0
HB-279 Luminespib 10.0
HB-243 Filanesib 10.0
HB-243 Filanesib 100.0
HB-279 Filanesib 1.0
HB-279 Filanesib 10.0
HB-279 Filanesib 100.0
HB-284 Luminespib 100.0
HB-282 Filanesib 10.0
HB-282 Filanesib 100.0

Supplementary Figure 4. Heatmap of cellular features measured in cell painting. Four HB models (HB-243, HB-279, HB-282 and HB-284) were treated with either 1 nM, 10 nM or 100 nM filanesib or luminespib or DMSO. HB-279 was also treated with 0.1 nM filanesib. Features were normalized plate-wise using standard scaling of the negative control samples (DMSO). The similarity of treatment responses was studied using data of all PDX models by hierarchical clustering with Euclidean distance metric.

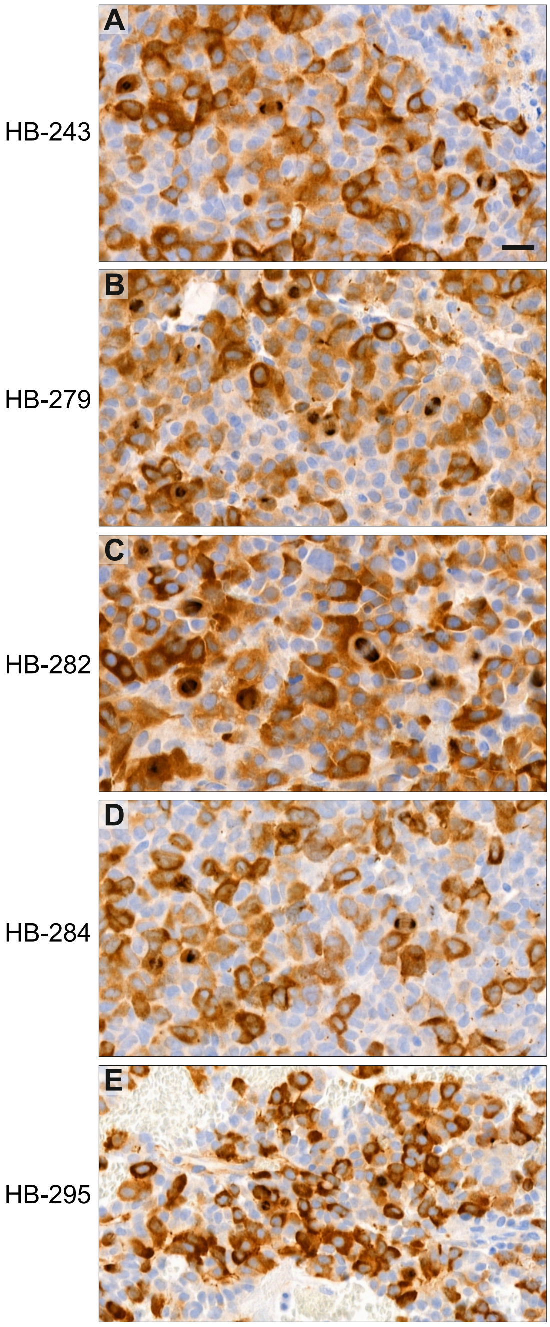

Supplementary Figure 5. Localization of KIF11 stain in the nuclei. In addition to tumor cell cytoplasm, the expression of KIF11 was localized in the nuclei of dividing cells that show mitotic spindle formation. This could be seen in all five HB PDX models. Magnification 40x, scale bar = 20 µm.

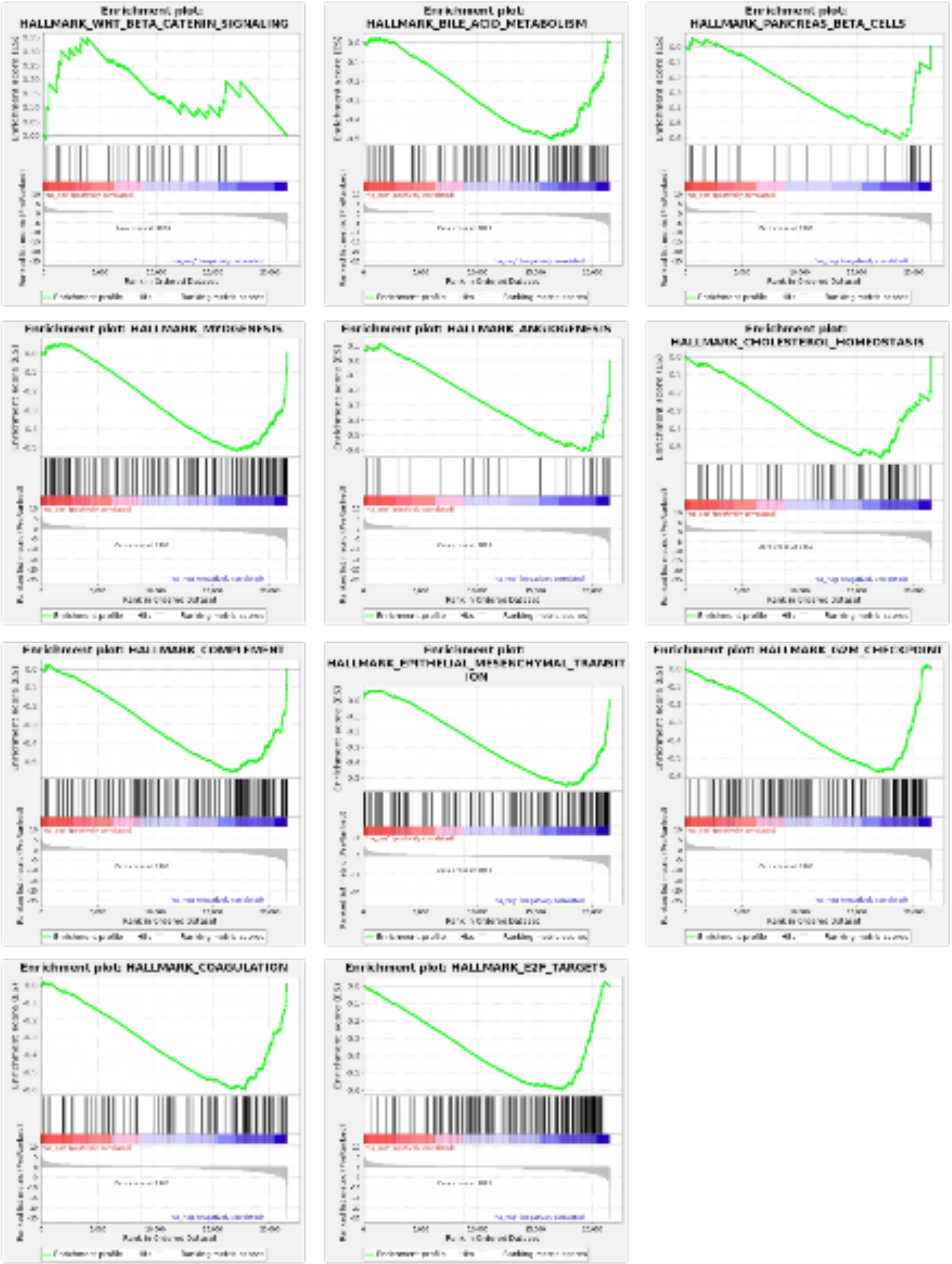

Supplementary Figure 6. Enrichment plots from the gene set enrichment analysis. A gene set enrichment analysis (GSEA) was performed to identify enriched gene sets among the differentially expressed genes between HB-279 and the other HB models. From Hallmark gene set collection, 18 gene sets with FDR ≤ 0.25 were found, of which 17/18 have lower expression in HB-279 when compared to the other models. Enrichment plots of top 10 lowest and highest expressed gene sets from Figure 7D are shown.

Supplementary Table 3.

Differentially expressed Hallmark Mitotic Spindle genes in HB-279 model. Hallmark Mitotic Spindle gene set consists of 200 genes. The differential expression of these genes in HB-279 versus the other five HB models was investigated. Among the 200 genes included in this gene set, 43 were significantly differentially expressed in HB-279. Of these 43 genes, 6 were higher and 37 lower expressed in HB-279. Genes were considered significantly differentially expressed with an adjusted p-value ≤ 0.1 for a minimum absolute log2 fold change of 0.6. Significantly higher-expressed genes are marked with orange and lower-expressed with blue.

ABI1	CDC42	FLNA	MYO9B	RICTOR
ABL1	CDC42BPA	FLNB	NCK1	ROCK1
ABR	CDC42EP1	FSCN1	NCK2	SAC3D1
ACTN4	CDC42EP2	GEMIN4	NDC80	SASS6
AKAP13	CDC42EP4	GSN	NEDD9	SEPTIN9
ALMS1	CDK1	HDAC6	NEK2	SHROOM1
ALS2	CDK5RAP2	HOOK3	NET1	SHROOM2
ANLN	CENPE	INCENP	NF1	SMC1A
APC	CENPF	ITSN1	NIN	SMC3
ARAP3	CENPJ	KATNA1	NOTCH2	SMC4
ARF6	CEP192	KATNB1	NUMA1	SORBS2
ARFGEF1	CEP250	KIF11	NUSAP1	SOS1
ARFIP2	CEP57	KIF15	OPHN1	SPTAN1
ARHGAP10	CEP72	KIF1B	PAFAH1B1	SPTBN1
ARHGAP27	CKAP5	KIF20B	PALLD	SSH2
ARHGAP29	CLASP1	KIF22	PCGF5	STAU1
ARHGAP4	CLIP1	KIF23	PCM1	STK38L
ARHGAP5	CLIP2	KIF2C	PCNT	SUN2
ARHGDIA	CNTRL	KIF3B	PDLIM5	SYNPO
ARHGEF11	CNTROB	KIF3C	PIF1	TAOK2
ARHGEF12	CRIPAK	KIF4A	PKD2	TBCD
ARHGEF2	CSNK1D	KIF5B	PLEKHG2	TIAM1
ARHGEF3	CTTN	KIFAP3	PLK1	TLK1
ARHGEF7	CYTH2	KLC1	PPP4R2	TOP2A
ARL8A	DLG1	KNTC1	PRC1	TPX2
ATG4B	DLGAP5	KPTN	PREX1	TRIO
AURKA	DOCK2	LATS1	PXN	TSC1
CEP131	DOCK4	LLGL1	RAB3GAP1	TTK
BCAR1	DST	LMNB1	RABGAP1	TUBA4A
BCL2L11	DYNC1H1	LRPPRC	RACGAP1	TUBD1
BCR	DYNLL2	MAP1S	RALBP1	TUBGCP2
BIN1	ECT2	MAP3K11	RANBP9	TUBGCP3
BIRC5	EPB41	MAPRE1	RAPGEF5	TUBGCP5
BRCA2	EPB41L2	MARCKS	RAPGEF6	TUBGCP6
BUB1	ESPL1	MARK4	RASA1	UXT
CAPZB	EZR	MID1	RASA2	VCL
CCDC88A	FARP1	MID1IP1	RASAL2	WASF1
CCNB2	FBXO5	MYH10	RFC1	WASF2
CD2AP	FGD4	MYH9	RHOF	WASL
CDC27	FGD6	MYO1E	RHOT2	YWHAE

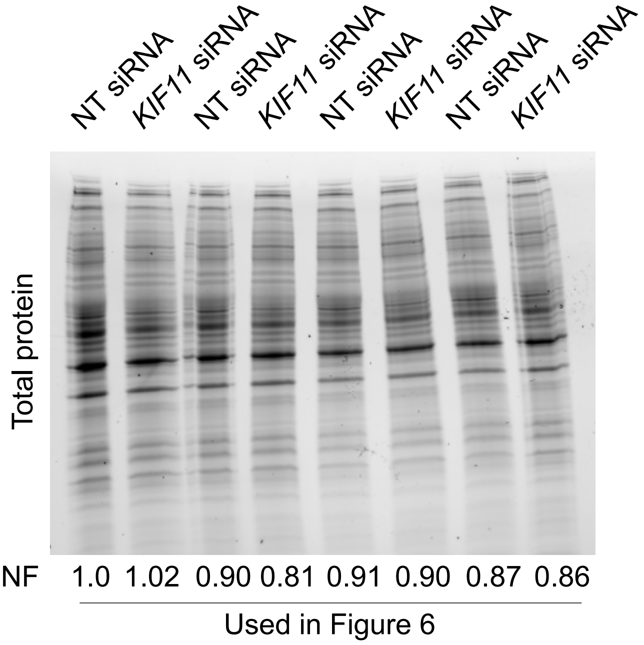

Supplementary Figure 7. Total proteins detected by StainFree technology. Normalization factor (NF) describes the amount of total protein in lane in relation to other lanes. NF = normalization factor.
